# Pesticides monitoring in surface water of a subsistence agricultural catchment in Uganda using passive samplers

**DOI:** 10.1007/s11356-022-22717-2

**Published:** 2022-09-08

**Authors:** Christelle Oltramare, Frederik T. Weiss, Philipp Staudacher, Oscar Kibirango, Aggrey Atuhaire, Christian Stamm

**Affiliations:** 1 Eawag: Swiss Federal Institute of Aquatic Science and Technology, 8600 Dübendorf, Switzerland; 2 Center for Primary Care and Public Health (Unisanté), University of Lausanne, 1066 Epalinges-Lausanne, Switzerland; 3 Department of Environmental Systems Science, ETH Zürich, 8092 Zurich, Switzerland; 4 Directorate of Government Analytical Laboratory (DGAL), Ministry of Internal Affairs, Kampala, Uganda; 5 Uganda National Association of Community and Occupational Health (UNACOH), Kampala, Uganda

**Keywords:** Smallholder farming, Surface water, Drinking water, Pesticides, Passive sampling, Environmental monitoring, Uganda, High-resolution mass spectrometry

## Abstract

Pesticides are intensely used in the agricultural sector worldwide including smallholder farming. Poor pesticide use practices in this agronomic setting are well documented and may impair the quality of water resources. However, empirical data on pesticide occurrence in water bodies of tropical smallholder agriculture is scarce. Many available data are focusing on apolar organochlorine compounds which are globally banned. We address this gap by studying the occurrence of a broad range of more modern pesticides in an agricultural watershed in Uganda. During 2.5 months of the rainy season in 2017, three passive sampler systems were deployed at five locations in River Mayanja to collect 14 days of composite samples. Grab samples were taken from drinking water resources. In these samples, 27 compounds out of 265 organic pesticides including 60 transformation products were detected. In the drinking water resources, we detected eight pesticides and two insecticide transformation products in low concentrations between 1 and 50 ng/L. Also, in the small streams and open fetch ponds, detected concentrations were generally low with a few exceptions for the herbicide 2,4-D and the fungicide carbendazim exceeding 1 ug/L. The widespread occurrence of chlorpyrifos posed the largest risk for macroinvertebrates. The extensive detection of this compound and its transformation product 3,4,5-trichloro-2-pyridinol was unexpected and called for a better understanding of the use and fate of this pesticide.

**Graphical abstract:**

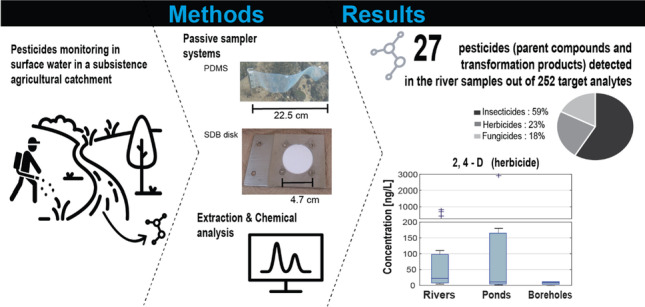

**Supplementary Information:**

The online version contains supplementary material available at 10.1007/s11356-022-22717-2.

## Introduction

Population increase has led to higher needs in food production, indirectly leading to increased pesticide use in agriculture to protect crops from unwanted pest infestation. Synthetic pesticides are used worldwide in conventional agriculture for pest control. The most common types of pesticides are herbicides, fungicides, and insecticides. Once applied in the field, pesticides may be mobilized by rainfall events (Leu et al. 2004; Doppler et al. 2012; de Souza et al. 2020) and can enter non-target environments and harm non-target organisms (e.g., aquatic organisms such as fish and invertebrates). Improper handling during and after application can also cause pesticides to end up in surface waters during dry weather (Rother 2018; Isgren and Andersson 2021).

Over the recent decades, many studies have documented pesticide pollution of different water bodies such as rivers (Moschet et al. 2015; Curchod et al. 2020; Postigo et al. 2021; Jayasiri et al. 2022), lakes (Papadakis et al. 2015; Merga et al. 2021; Satiroff et al. 2021), and groundwater (Loos et al. 2010; Lopez et al. 2015). However, the majority of these monitoring and research efforts have focused on the Global North (Jurado et al. 2012; Petrie et al. 2015; Sousa et al. 2018; Daam et al. 2019). Thus, knowledge about pesticide levels in water bodies in many tropical low- and middle-income countries (LMICS) is limited compared to Europe or North America. This leads to important knowledge gaps on pollution in tropical settings. Indeed, the differences in temperature, sunlight, or amount of rainfall could influence the concentration found in the water bodies (Lewis et al. 2016; Op de Beeck et al. 2017; Vela et al. 2019; Taylor et al. 2020). Moreover, the type of farming also influences pest management and, therefore, the use of pesticides.

Smallholder farming is the most common type of farming in LMICS (Staudacher et al. 2020; Isgren and Andersson 2021). This farming system can be vulnerable to environmental pesticide contamination and the associated negative impacts on human and environmental health. Intense urban farming close to headwater streams and rivers can cause high pesticide contamination due to the direct transfer to streams (Branchet et al. 2018). Where smallholder farmers have limited awareness and formal education, the risks of potentially harmful effects for farmers and the environment during pesticide applications increase (Wiedemann et al. 2022). The available data show that in urban centers or settlements with poor sanitary infrastructures, the contamination is higher and could cause high environmental risk (K’oreje et al. 2020). Moreover, most African countries are using several different pesticides. Due to a lack of regulation or illegal practices, even some banned pesticides are used. Moreover, data showed a significant increase in the value of pesticides imported into Uganda within the last 20 years (Andersson and Isgren 2021). However, data on pesticide occurrence in the aquatic system are still limited in African countries (K’oreje et al. 2020). Monitoring data, for example, indicate that most organochlorine pesticides might still be used in Tanzania or Kenya (K’oreje et al. 2020; Olisah et al. 2020).

In Uganda, farmers tend to purchase pesticides from informal (often) non-certified suppliers, who, in most cases, do not offer information regarding proper pesticide handling (Andersson and Isgren 2021; Staudacher et al. 2021). Practices such as over-dosage, inappropriate cleaning of spray equipment and clothing, indiscriminate disposal of pesticide containers, and spraying close to waterways or water sources are frequently observed (Matthews 2008; Staudacher et al. 2020). These practices could lead to contamination of the surface and groundwater.

An assessment of pesticide pollution in waterbodies requires considerable infrastructure and resources for sampling and subsequent chemical analysis. Expensive automatic samplers often used in the Global North may not be available or difficult to use in remote locations or areas with little infrastructure and security problems. For these reasons, the available information in many tropical LMICS on pesticides in aquatic systems relies mostly on grab samples representing only limited moments in time (Bernard et al. 2019; Valenzuela et al. 2020). Passive sampling can overcome such limitations and provide time-averaged concentrations over days or a few weeks. Different systems exist for such a passive approach including membrane-based systems (Vrana et al. 2005; Ahrens et al. 2015; Booij et al. 2016; Taylor et al. 2020) or collection devices for water samples (Schönenberger et al. 2021). Passive samplers permit a relatively easy deployment because they require hardly any additional infrastructure. These small devices allow application in a situation with limited space for equipment (e.g., small stream with a narrow riverbed) or difficult access (e.g., far from roads) (Vrana et al. 2005; Estoppey et al. 2014; Ahrens et al. 2015; Schreiner et al. 2020). Passive sampling has proven to be a cost-effective and robust method to analyze a wide range of chemical compounds, including pesticides (Moschet et al. 2015; Curchod et al. 2020; Taylor et al. 2020).

This paper aims at filling knowledge gaps about pesticide occurrence in African tropical water bodies in the context of smallholder farming by studying an agricultural watershed north-west of Kampala, Uganda, where smallholders constitute the majority of the population (Isgren and Andersson 2021). The specific study’s objectives were (i) to quantitatively assess the pesticide occurrence of a large set of compounds in River Mayanja and selected headwaters, (ii) to analyze the spatio-temporal patterns of detected pesticides, and (iii) to evaluate the contamination of different drinking water sources.

## Methods

### Study area

**Fig. 1:**
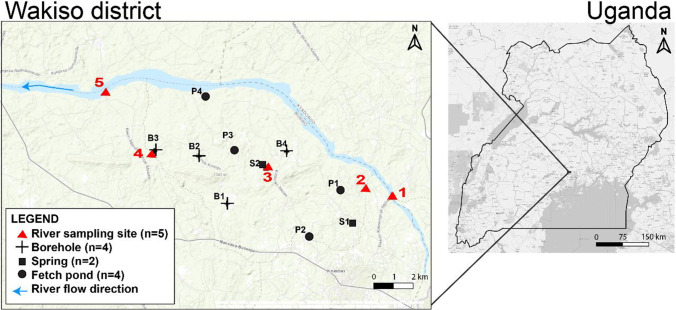
Map of the sampling area located in Central Uganda, (  : river site sampling location,  B: boreholes, ■S: springs, and ●P: fetch ponds). Source: OpenStreetMaps, Qgis

The field campaign focused on a crop farming area in Wakiso District (0°38 N and 32°48 E), Uganda (Fig. 1). The climate of the study site is tropical, with annual average precipitation of 1470 mm/year observed between 2000 and 2016 (World Bank Groups 2021), with two rainy seasons: one typically between March and May and the other between September and November. The altitude ranges between 1100 and 1300 m above sea level. Farmers in the area are predominantly smallholders operating conventional subsistence farming, growing crops such as tomatoes, cabbage, sweet potatoes, cassava, bananas, and coffee for home consumption and sale in the local markets (Atuhaire et al. 2017; Staudacher et al. 2020). Short-season (3–4 months) crops such as tomatoes and cabbages are mostly grown for commercial purposes and are intensively treated with insecticides and fungicides. With the exception of the first month after planting, these synthetic pesticides are applied to these horticultural crops almost at least once a week for the rest of the season (Atuhaire et al. 2017). On the other hand, pesticides are rarely used on long-season crops such as banana and cassava, with the exception of coffee which is occasionally sprayed majorly with insecticides. In short-season crops, herbicides are generally used for short periods, mainly in field preparation before planting. With regard to long-season crops, herbicides are occasionally spot applied to control weeds at the edges and inside the fields of coffee, for instance.

The locations of the different sampling points investigated in River Mayanja and three of its tributaries are illustrated in Fig. 1 (Strahler classification: Mayanja: 3#). River Mayanja flows in a north-western direction from Wakiso draining into River Kafu (Onyutha et al. 2021). The sampling points were spread across two sub-counties of Mende and Masulita (exact location on Supplementary Table S1) close to arable land or wetland as they were defined as the major land use (Emerton et al. 1998). The three tributaries correspond to small headwater streams. Discharge varied during the study period between 0.05 m/s and at least 0.36 m/s (onsite measurements, see below).

Drinking water sources were investigated at ten sampling points (Fig. 1) by three replicates of grab samples over the three months of investigation (September to December 2017). The drinking water sources (Supplementary Table S1) represented three types: springs, boreholes, and fetch ponds, which were mostly fed by surface water.

### Sampling

The study was conducted between September 15th and December 1st, 2017. Stream sampling was carried out with three different passive sampling devices. First, membrane-based passive samplers were deployed at each river sampling station for two-week sampling periods. Two different membrane materials were used to collect pesticides with a large spectrum of physico-chemical properties: polydimethylsiloxane (PDMS) membranes (shielding solutions, thickness: 0.25 mm) for nonpolar compounds and reverse phase sulfonated styrene–divinylbenzene (SDB) disks (Empore™, Modell 2241, 47 mm, thickness: 0.5 ± 0.05 mm, 3 M) for (semi-)polar compounds. SDB and PDMS were installed on stainless steel holders as described by Moschet et al. (2015) and Vermeirssen et al. (2009). Second, a water-level proportional sampling bottle system (WLP) was used to collect continuously water for periods of one week. The WLP bottle system continuously samples water depending on the water level with the help of a precision valve (Göldi Präzisionsmechanik AG, Switzerland) as a resistant controlling outflow of air out of the system and inflow of water. This enables a time-integrated and water level-weighted quantification of chemicals. The working principle of the WLP bottle system is based on the continuous intrusion of water into a collecting bottle, where the volume sampled per unit of time is dependent on the hydrostatic pressure. The working principle is described in more detail by Schönenberger et al. (2021) and Weiss (2021). The samples were stored at river temperature until the bottles were exchanged. The target volume was 0.6 L per week in order to mix two samples and get a 14-day (14-d) composite sample representing the same period as the membrane-based samples. The samples were transported to the lab in Kampala, Uganda, and stored at 4 ℃ until further processing.

At each site, a HOBO® U20L Water Level Data Logger (ONSET, Switzerland) was installed to measure the water level every 15 min. At some tributary sites (sites 2 and 3), an automatic camera (DÖRR Snapshot, Germany) took pictures to observe the flow dynamic of these small streams with the same temporal resolution. In the outflow of the catchment in the Mayanja (site 5), it was not possible to get data for the water level because we could not safely access a location allowing a stable installation in the river.

#### Membrane-based passive samplers

The PDMS sheets were applied according to Moschet et al. (2014). Briefly, the PDMS were cut into pieces of 22.5 × 10.0 cm and conditioned by Soxhlet in ethyl acetate for 100 h. After conditioning, the sheets were stored in methanol at room temperature. Upon removal, the PDMS sheets were rinsed in the river to remove the biofilm and then stored in an amber glass vial at − 20 ℃ until analysis in Switzerland. A piece of 10 × 10 cm was used for the extraction; the remaining piece was kept for quality control. The extraction was performed using accelerated solvent extraction (ASE, Dionex ASE 350, Switzerland) with the following parameters: five extraction cycles, each with methanol at 120 ℃, a static time of 10 min, a rinse volume of 75%, and a purging of 110 s. The extract was spiked with internal standards (Supplementary Table S2) and subsequently evaporated with an automated evaporator system until dryness (Büchi Syncore® Analyst, Switzerland) at 50 ℃ 159 mbar and 300 rpm (ca. 4.37 RCF). The sample was reconstituted with 0.5 ml of hexane. The clean-up phase was performed using a Pasteur pipette filled with the first layer of 500 mg of silica gel (silica gel 60, 0.063–0.2 mm, Merck Germany, activated at 130 ℃ for 5 days) separated by a frit with a second layer at the bottom of 500 mg of Isolute C18 (Biotage, USA). After conditioning the column with 6 ml of hexane, the sample extract was loaded. Subsequently, 2 ml of hexane was used to rinse the column. The elution was performed by adding 10 ml of acetonitrile. We used the automated evaporator system (ACROS organics, Switzerland) to evaporate the eluate to dryness at 50 ℃, 117 mbar, and 300 rpm. Finally, the sample was reconstituted with 1 ml of hexane, transferred to a centrifuge vial, and centrifuged at a speed of 4000 rpm for 30 min. The supernatant was transferred in a 2 ml screw cap vial to analyze.

The Chemcatcher® contained an Empore SDB-RPS disk and a polyethersulfone (PES) membrane (Ø 47 mm, pore size: 0.45 µm, Supor, PALL, Switzerland). They were prepared according to Vermeirssen et al. (2009). Briefly, the SDB and the PES were conditioned in methanol for 30 min, followed by a conditioning step using filtered water, as well for 30 min. Then, the SDB disk was installed on the iron holder and coved by a PES membrane. The holders with the passive samplers were stored at room temperature in filtered water until their deployment in the field. At each sampling site, two replicates were installed: One was analyzed, and the second was used as a backup. The SDB disks were stored individually in an amber glass vial at − 20 ℃ until analysis. In the lab in Switzerland, the extraction was conducted as described by Moschet et al. (2013). Briefly, 6 ml of acetone was added to each glass vial containing the SDB disk at room temperature. The vials were shaken for 30 min. Afterward, the acetone extract was transferred into individual centrifuge tubes, and internal standards (Supplementary Table S3) were added and evaporated to 1 ml under nitrogen flow. Each SDB disk was extracted for a second time using 5 ml of methanol and was shaken for 30 min. Then, the methanol extract was mixed with the concentrated acetone extract. The 6 ml total extract was filtered using a polytetrafluoroethylene (PTFE) filter and then evaporated to a volume of approximately 0.1 ml. Finally, the extract was adjusted to 1 ml with nanopure water. The samples were centrifuged and transferred into a screw cap vial for analysis.

#### Water samples (grab samples and WLP samples)

The samples from the different drinking water sources and the WLP samples from the rivers were concentrated using a three-layered (Envicarb, Strata-X-AW Phenomenex:Strata-X-CW Phenomenex:Isolute ENV1 Biotage, Oasis HLB) SPE-cartridge type Silovo as described by Kern et al. (2009) and Volger (2013). The cartridges were packed using a 6 ml empty cartridge. The detailed cartridge composition is listed in Supplementary Text S1.

Before loading the cartridges with the 1 L water samples collected in the field, the cartridges were preconditioned by adding firstly 1 ml of ammonium acetate (1 M) and then adjusting the pH to 6.5–6.9 with ammonia or formic acid. The sample was then filtrated through a 0.7 µm glass filter (GF-F, Whatmann, Switzerland). Finally, the internal standards were added (Supplementary Table S3). The cartridge was conditioned using 5 ml of methanol and then 10 ml of filtered water. The samples were loaded onto the cartridge at a constant flow rate (~ 8 ml/min). Once the sample volume of 1 L was loaded, the cartridges were dried and stored at − 20° until elution in Switzerland. The cartridges were eluted back flush to avoid the sorption of all analytes on the layer of Envicarb. The elution was performed with 6 ml of methanol/ethyl acetate (50:50) containing 2% ammonia, then 3 ml of methanol/ethyl acetate (50:50) containing 1.7% of formic acid, and finally 2 ml of methanol. The extracts were concentrated to a final volume of 0.1 ml under a gentle stream of nitrogen. Nanopure water was added to reach a final volume of 1 ml. Samples were centrifuged at 4000 rpm during 30 min, and the supernatant was transferred to a screw cap glass vial for analysis.

### Analysis and target substances

The extract from the PDMS sheets was measured using gas-chromatography coupled to a triple Quadrupole (GC–MS/QQQ) (Moschet et al. 2014). The column was a Zebron ZB-5MS (15 m, 0.225 mm inner diameter, film thickness 0.25 μm, Phenomenex, Switzerland). The carrier gas (He) was set at a constant flow of 1.2 ml/min. The ionization mode was set to positive electron ionization (EI). The best transition was used as a quantifier and the second as a confirmation ion (qualifier). The 17 target compounds and their respective LOQs are listed in Supplementary Table S2.

The extracts from SDB disks and the SPE extract were measured by high-performance liquid chromatography high-resolution mass spectrometry (HPLC-HR MS/MS) (Moschet et al. 2015). Methanol and nanopure water (both acidified with 0.1% formic acid) were used as the eluents for the gradient. Chromatographic separation was achieved using an XBridge C18 column (3.5 µm, 2.1 × 50 mm; Waters, Switzerland) with a pre-column (2.1 × 10 mm, Waters, Switzerland). The HPLC was connected to an electrospray ionization (ESI) source of a QExactive plus mass spectrometer (Thermo Fisher Scientific, Switzerland) which triggered MS2 spectra. Each sample was analyzed in positive and negative ionization modes separately. The target list of 250 compounds and the internal standards (Supplementary Table S3) is similar to the work published by Curchod et al. 2020. With this list, we focused on a wide range of modern pesticides, which are mostly rather polar compounds. The limits of quantification (LOQ) for each compound and sample type are listed with the calculated recoveries in the same Supplementary Table S3.

### Meteorological data

To obtain local precipitation data, selected contact farmers were guided to record daily rainfall readings on a graduated scale rain gauge installed in their gardens for the entire sampling period.

### Pesticide use data collection

Information about pesticide use was collected during an observational cross-sectional study between September and November 2017 (Staudacher et al. 2020). That study was done in parallel with the water sampling campaign. Using a structured questionnaire, data was gathered from farmers in the catchment area regarding the 15 pesticide-active ingredients most commonly used in the previous 12 months. The list of active ingredients was compared only after having the results to avoid any bias results (e.g., influencing the questionnaire to the farmers).

### Data analysis

Membrane-based passive sample results were evaluated in terms of mass per samplers. The actual water concentration ${C}_{\mathrm{water}}$ was estimated by dividing the mass on the membrane ${M}_{\mathrm{membrane}-\mathrm{based sampler}}$ with the compound-specific sampling rate ${R}_{s}$ and the sampling period $t$ as shown in Eq. (1):

1 $${C}_{\mathrm{water}}=\frac{{M}_{\mathrm{membrane}-\mathrm{based sampler}}}{{R}_{s}\times t}$$

We considered the sampling rate available from published literature for the SDB disks (Moschet et al. 2015; Ahrens et al. 2015; Charriau et al. 2016). For the PDMS sheets, we used the average sampling rate ${R}_{s}$ of 35 L/d estimated by Moschet et al. (2014) normalized for 600 cm^2^ of rubber sheet. Therefore, we used 35/6 L/d as the sampling rate in the estimation of the aqueous concentration.

In order to assess the ecotoxicity of the pesticide levels in a consistent way across compounds, the concentrations were compared to Environmental Quality Standards (EQS) that have been derived according to a European Technical Guidance document (European Commission. Directorate General for the Environment 2011). The EQS values define the concentration threshold below which no adverse effect on aquatic organisms should occur (European Commission Directorate General for Health and Food Safety 2018). To evaluate the risk assessment for drinking water, values from the East African Community standard (East African Community 2018) in conjunction with WHO guidelines (World Health Organization 2017) were compared, as they are the standard values used in Uganda.

## Results and discussions

### Pesticides in the river and streams

#### Weather conditions

**Fig. 2:**
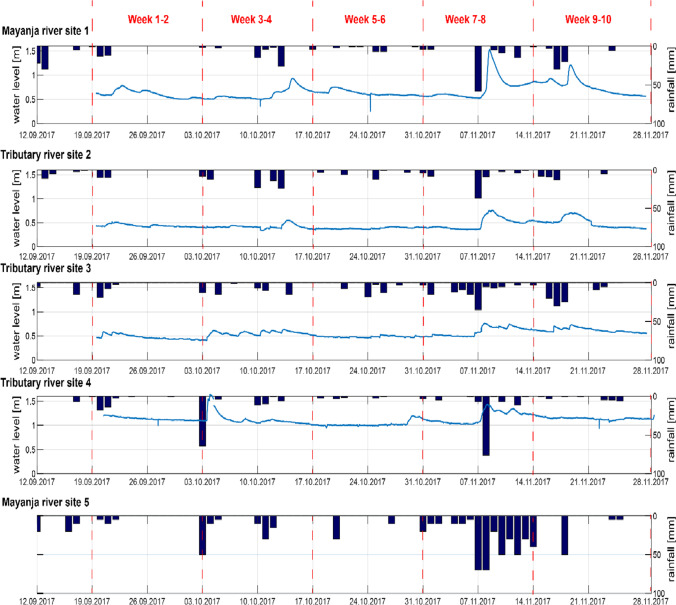
Water level and rainfall at each sampling site throughout the sampling campaign in the two main streams of River Mayanja and three of its tributaries. On site 5, no water level was recorded. The left *y*-axis corresponds to the water level (blue line) and the right y-axis to the rainfall (bars plotted inverse)

Rainfall and associated surface runoff, considered a major driver for the transport of pesticides to streams, varied strongly in space and time during the study. We measured between 0.5 and 76 mm per day of rainfall during the entire sampling campaign, equivalent to 12 to 405 mm within 14 days of sampling intervals. Figure 2 illustrates that some of the rain events occurred across the entire area while others only affected part of the catchment. For example, 50 mm was recorded on 3.10.2017 at site 5, whereas nearly no (< 2 mm) rain was recorded at the other site of the catchment area (site 1). During some weeks (19.09.2017–3.10.2017), hardly any rainfall was observed at any site, while during the weeks 31.10.2017–14.11.2017, wet conditions were observed everywhere (Fig. 2).

The stronger rain events caused a direct discharge response as reflected in the water level measurements (Fig. 2). Field observations such as the automated photographs showed that discharge repeatedly exceeded bankfull conditions and spread on the field (see Supplementary Fig. S1 illustrating the dynamic with pictures from site 2). Therefore, stream water may have directly reached parts of fields that had previously been treated with pesticides.

#### Pesticide occurrence

**Fig. 3:**
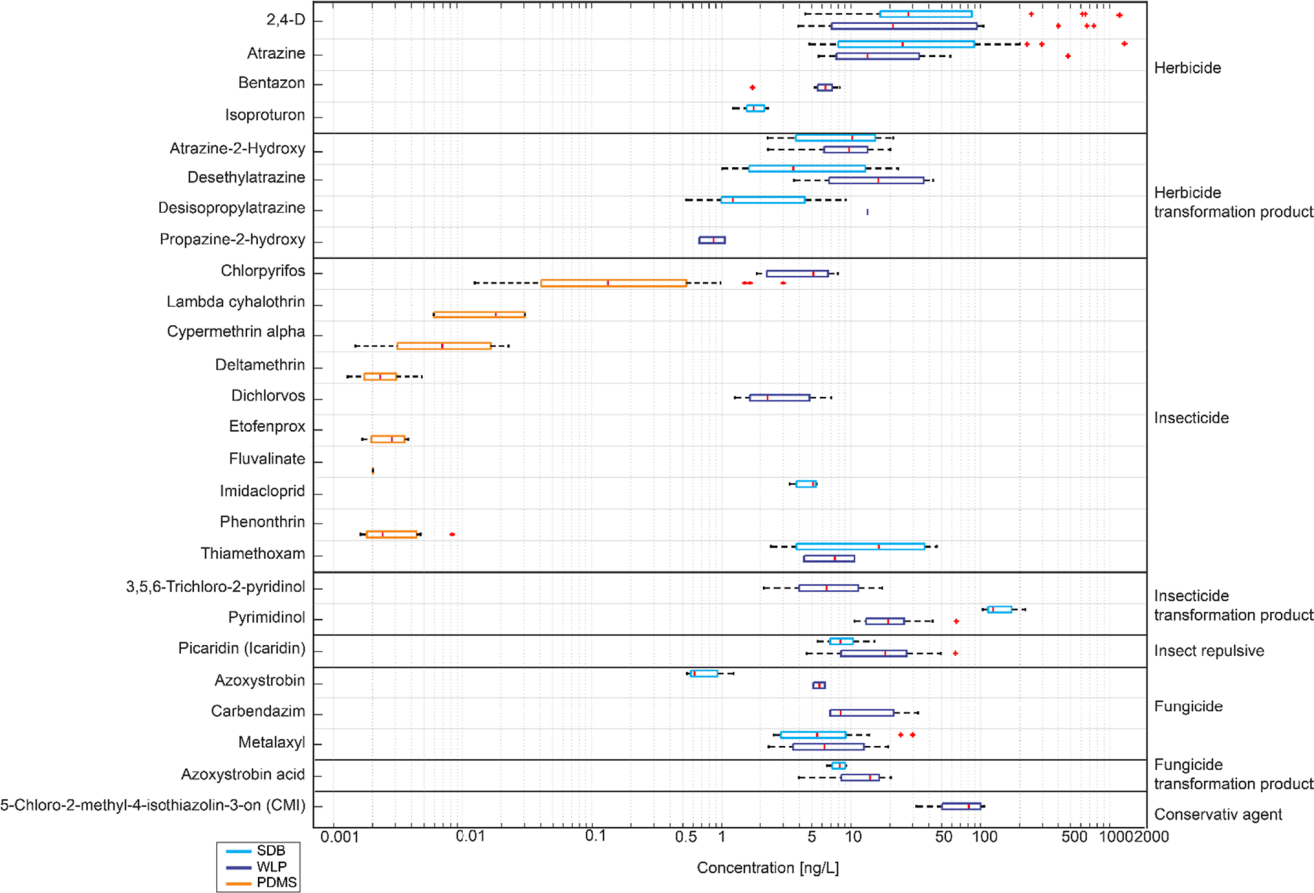
Pesticide concentrations quantified (above the limit of quantification) from the three sampling methods: SDB disks, WLP, and PDMS

In the SDB and the WLP samples, 13 and 18 compounds out of the 250 target substances were quantified above the LOQ, respectively (Fig. 3). The eleven parent compounds included similar numbers of herbicides (4), insecticides (4), and fungicides (3). Six additional insecticides (pyrethroids) were detected on the PDMS sheets (Fig. 3). The detected transformation products (TPs) originated from herbicides (4), insecticides (2), and one fungicide. We also found the insect repellent picaridin, while N,N-Diethyl-m-toluamide (DEET) had to be excluded from the analysis due to high blank values. This problem has been repeatedly reported in the literature (Ferguson et al. 2013; Marques dos Santos et al. 2019) and was probably caused by the frequent DEET use in daily life by laboratory and field workers.

The split between detects of herbicides, insecticides, and fungicides across all three sampling devices revealed a predominance of insecticides (*N* = 10; Fig. 3) and a similar share of herbicides and fungicides. In African studies, insecticides were detected in a similar percentage as herbicides, for example, in South Africa, where 18% were insecticides and 18% were herbicides (Rimayi et al. 2019). In a systematic review on pesticide use in Malawi, insecticides were applied mainly in field crops like Maize (Soko 2018). These findings reflect the larger use of insecticides in tropical countries during the rainy season (Añasco et al. 2010; Carazo-Rojas et al. 2018; Sharma et al. 2019; Jayasiri et al. 2022; Weiss et al. (submitted)). The rainfall has a direct effect on the compound transport and dilution, depending on the last application and the dose (Hrachowitz et al. 2016; Jayasiri et al. 2022). Furthermore, the high occurrence of insecticides during the rainy season could correspond to the higher use due to specific pests when the crops are mature (Añasco et al. 2010). Unfortunately, we can hardly compare our results with studies from other African tropical countries since they mostly focused on OCPs (Ntow 2001; Mmualefe et al. 2009; Okoya et al. 2013; Ogbeide et al. 2015; Teklu et al. 2016). A recent study from Tanzania (Materu et al. 2021) screened for almost 100 pesticides and detected 11 compounds including six herbicides, two insecticides, and three herbicide transformation products.

Due to different crops and pest pressure, studies from temperate regions (in the Global North) often show that herbicides dominate pesticide exposure (Spycher et al. 2018; Taylor et al. 2021) compared to tropical countries.

The SDB and the WLP samples provided partially complementary information on pesticide occurrence. Most compounds (*N* = 11) were detected in both samplers, two compounds only with the SBD disks and seven only in the WLP (Fig. 3). Chlorpyrifos was the only compound detectable with all three sampling methods. However, there was no concentration above the LOD with the SDB sampling. In most cases, concentration levels below the LOQ of the other systems explained why pesticides were only detected in one sampler. The exception was carbendazim, which was only quantifiable in the WLP samples even though the limit of quantification was higher than the respective LOQ of the SDB method (Fig. 3). The reason for this is not clear. Schreiner et al. (2020) tested similar SDB material in controlled experiments and could detect carbendazim. Moreover, other studies using SDB disks in natural environments detected carbendazim (Mutzner et al. 2019; Pinasseau et al. 2020; Schreiner et al. 2020) as well. Therefore, we should exclude the hypothesis that carbendazim does not adsorb on the SBD disk or is not extracted properly with the method used in this study.

Our analytical methods covered about 44% of all currently registered synthetic pesticides in Uganda (50 out of 113 compounds, see Supplementary Table S4) plus about 140 additional pesticides that are used in many countries. The detected pesticides though were all listed in the Agricultural Chemicals Register for Uganda (Ministry of Agriculture, Animal Industry and Fisheries (MAAIF) 2017). Hence, we have no indication of the use of illegal compounds. At the local level, our analytical window covered 11 out of 15 specific active ingredients that the farmers remembered as having used during the last 12 months, according to Staudacher et al. (2020) (see Table Supplementary S5). The two most used pesticides in our study area (glyphosate and mancozeb) (Staudacher et al. 2020) were not included in our study since their detection was not feasible with the applied analytical methods.

In addition to the pesticides with reported local use, we detected another set of 19 pesticides. One of these compounds was atrazine, which was found in more than 70% of the environmental samples. This is probably due to the high persistence of the compound in the subsurface, causing its detection in many areas long after its ban (Kiefer et al. 2019). However, atrazine is still allowed in two products in Uganda (Ministry of Agriculture, Animal Industry and Fisheries (MAAIF) 2017). It could still be used by farmers but may not be reported due to lack of knowledge since the registered product is a mix with metolachlor.

A surprising discrepancy was found for the insecticide chlorpyrifos. It was detected in all the samples but was reported to be rarely used by farmers (Staudacher et al. 2020). One possible cause could be that chlorpyrifos was commonly used on livestock as an acaricide and sprayed weekly, which was not reported in the questionnaire. Furthermore, chlorpyrifos is known to strongly volatilize, contributing to the widespread distribution of the compound (Laabs et al. 2002).

The frequent detection of atrazine and chlorpyrifos fits to observations from the literature that have highlighted the widespread contamination in Africa with these pesticides in surface water (Lehmann et al. 2018; Curchod et al. 2020) or groundwater (Sorensen et al. 2015).

Some mismatch between reported use and pesticide detection in water bodies was revealed when comparing our results with another study located in the Wakiso district. Atuhaire et al. (2017) analyzed the level of dithiocarbamate residues on tomatoes. They also reported that mancozeb was the most used pesticide on this kind of crop, followed by products using combining profenofos and cypermethrin, abamectin and acetamiprid, or metalaxyl and mancozeb. Despite the frequent use of acetamiprid on tomato crops, this compound was not found in the rivers in the study area, although tomatoes are one of the plants cultivated by smallholder farmers. Acetamiprid was detected in less than a third of the drinking samples. Crops such as tomatoes and cabbages are mostly grown for commercial purposes and are intensively treated with insecticides and fungicides. Herbicides are generally used for short periods, mainly in field preparation before planting or occasionally applied to control weeds at the edges and inside the fields of coffee (Atuhaire et al. 2017).

The concentration ranges of all 26 detected pesticides including transformation products for the 14-d composite samples differed strongly between the more apolar insecticides and the more polar compounds (Fig. 3). The insecticide concentrations on the PDMS sheet were mostly in the sub-ng/L range with some exceptions for chlorpyrifos. The pesticides and transformation products detected with the SDB and WLP samplers ranged mostly between 1 and 100 ng/L. For the two herbicides, 2,4-D and atrazine, a few samples yielded concentrations of several hundred ng/L and even reached 1.3 µg/L for 2,4-D.

The concentration ranges for the 10 compounds detected in both the SDB and WLP samplers (Fig. 3) were mostly similar with a few exceptions (Supplementary Fig. S2 and Table S6). Given that the two systems were deployed during the same periods, this offers the possibility to directly compare the values. For the eight compounds with sufficient data pairs for comparison, the median of the fold difference was 1.8 and the average difference was 2.3. The maximum deviation was a factor of five. These findings are in line with the uncertainty of the literature values of one order of magnitude (Curchod et al. 2020). There was no systematic difference when comparing the concentration found in SDB versus WLP. The concentration of picaridin found in the WLP was always higher than the ones found in the SDB. On the contrary, the concentration of atrazine or metalaxyl was higher in the SDB samplers.

Additionally, with the quantification of compounds with SDB disks and the WLP sampler, one can compare these data to evaluate the SDB sampling rates from the literature (Supplementary Fig. S2). The ratios of empirically estimated sampling rates from our data and literature values (Ahrens et al. 2015; Curchod et al. 2020) have a median of 1.1 and range between 0.2 and 3.9 (Supplementary Table S6). Despite the different sampling principles (time-proportionally (SDB) versus water-level proportional (WLP)), these values provide evidence that the reported concentrations are plausible.

For chlorpyrifos, the concentrations in the WLP could also be compared to those from the PDMS. Here, no clear relationship could be found (Supplementary Fig. S3). The concentration levels were clearly lower from the PDMS sheets (Fig. 3). A very similar finding was found in a different field study in Costa Rica using the same set of passive sampling devices (Weiss et al. (submitted)). This could suggest that the assumed sampling rate for chlorpyrifos was too low for the prevailing sampling conditions. The sampling rate for this compound is assumed to be similar to PCBs and PAHs (Moschet et al. 2014). It remains open whether these deviations can be explained by factors like the flow velocity or the biofilm on the samplers.

**Fig. 4:**
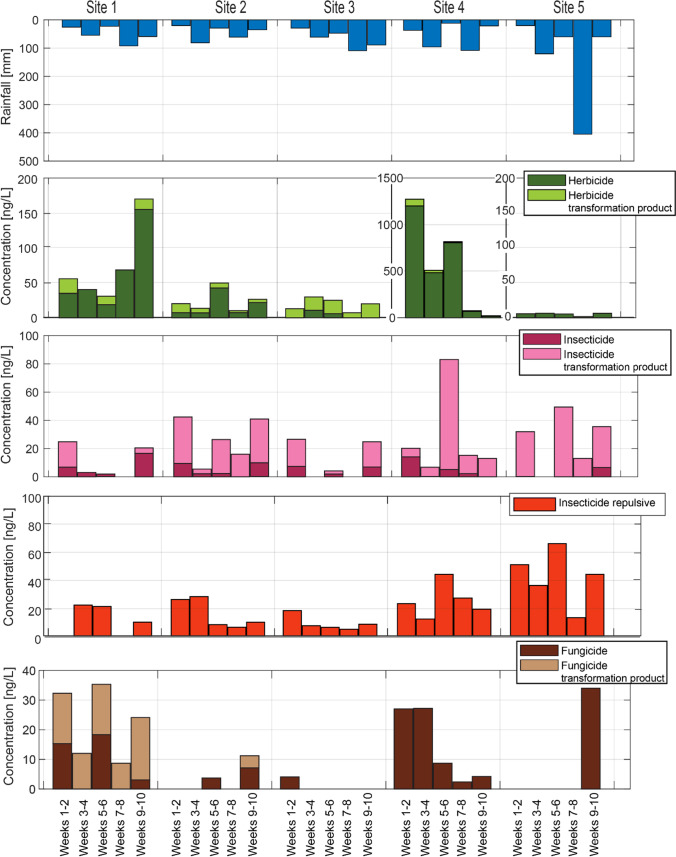
Sum concentration (in the WLP samples) through the different sites and time with biweekly rain data (added up over 14 days)

The different compounds revealed various spatio-temporal patterns. The herbicide levels differed markedly between the sites as well as in time. At site four, the highest concentrations were observed, exceeding 500 ng/L for six weeks in a row (Fig. 4). During the following four weeks, however, only low levels were observed (< 70 ng/L). Also, at site 1, there were marked temporal differences in herbicide concentrations. The temporal changes did not reveal any consistent temporal pattern with rainfall or discharge. This suggests that the concentration levels were rather driven by recent applications in the respective catchment. Chlorpyrifos was found in all sites and at each time point in the PDMS sheets (Supplementary Fig. S4). This demonstrates widespread contamination that sometimes reached high concentrations relative to the toxicity of the compound (discussed *below*). Because of chlorpyrifos’ short half-life, less than 30 days in water (Solomon et al. 2014), these results point to regular use of the compound throughout the study area.

**Fig. 5:**
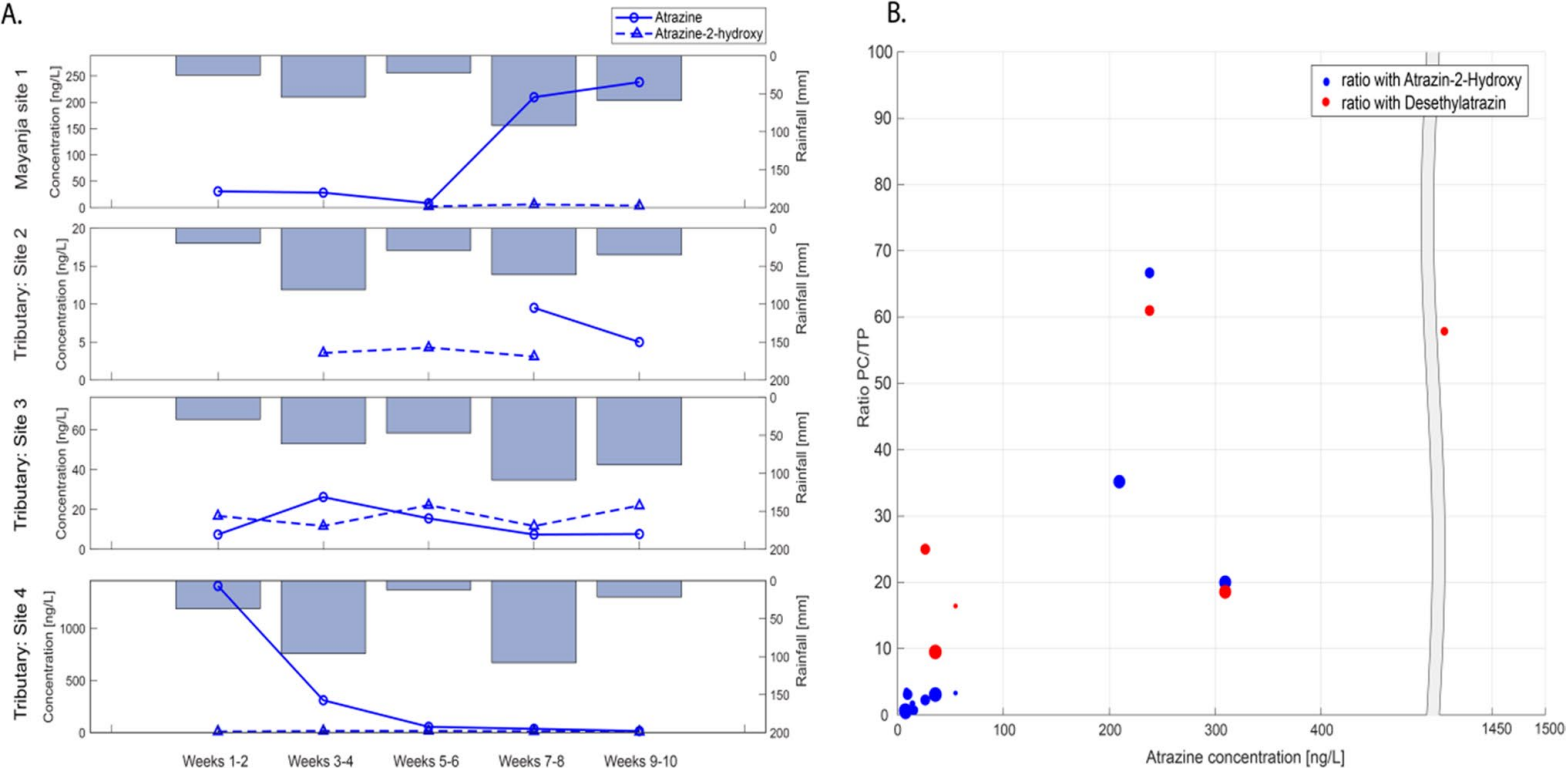
(**a**) Relationship between the atrazine and atrazine-2-hydroxy concentration from the SDBs, at the different sampling points with regard to rainfall. (**b**) Relationship between the atrazine and the ratio of the parent compound (PC) over the transformation product (TP); the size of the marker indicates the amount of rain for the respective two weeks of sampling

Interestingly, the ratios between parent compounds (PC) and transformation products (TP) varied substantially between herbicides and insecticides (Fig. 4). For the herbicides, the parent compounds dominated the sum concentrations at four of the five sites, while it was the opposite for the insecticides. This was mainly caused by the widespread presence of one transformation product of chlorpyrifos (3,4,5-trichloro-2-pyridinol). At all sites, this transformation product had the larger share of the concentration sums. Figure 5b illustrates ratios between atrazine and its transformation products (atrazine-2-hydroxy and desethylatrazine). As the concentration of atrazine increases, the ratio of PC over TP increases as well. Unfortunately, there were not enough samples in the higher concentration range (> 50 ng/L) to conclude to a linear relationship. Figure 5a compares the concentration of atrazine and its transformation product (atrazine-2-hydroxy) in time related to the amount of rain over the different sites. No relationship could be detected.

### Ecotoxicological risk assessment

To assess the ecotoxicological risk in the streams, we relied on EQS that have been derived in a consistent way across all compounds (Supplementary Table S7) (ecotoxcenter, EAWAG/EPFL 2020). In Uganda, these EQSs do not have legal relevance because no corresponding quality standards are defined (Ministry of Water and Environment, Republic of Uganda 2013). Therefore, this risk assessment provides mainly a relative comparison indicating the most critical compounds from an ecotoxicological point of view.

We determined the Chronic Risk Quotients by using the 14-d composite sample data and chronic EQS values. These values were exceeded most prominently by the insecticide chlorpyrifos detected in WLP samplers. The data exceeded the chronic EQS of 0.46 ng/L up to 17-fold. The second compound exceeding an EQS was the herbicide 2,4-D in one sample. Overall, the results suggest that the macroinvertebrates were the organisms most at risk (Supplementary Fig. S5).

The evaluation of the ecotoxicological risk was based on the EQS values of the parent compound. There are hardly any EQS values for transformation products. In the river samples, the main compound causing risk is chlorpyrifos, as discussed above. However, actual knowledge indicates that the toxicity of TCP should not be underestimated (Echeverri-Jaramillo et al. 2020). Furthermore, the properties of TCP tend to make this compound more critical because it is more mobile and more persistent (Zhao et al. 2017). In the environment, exposure to chlorpyrifos and TCP can induce a lethal response at low concentrations for bacteria and algae (Echeverri-Jaramillo et al. 2020). This example shows the importance to monitor parent compounds as well as their transformation products and to assess the toxicity of the mixture.

### Pesticides in drinking water resources

**Fig. 6:**
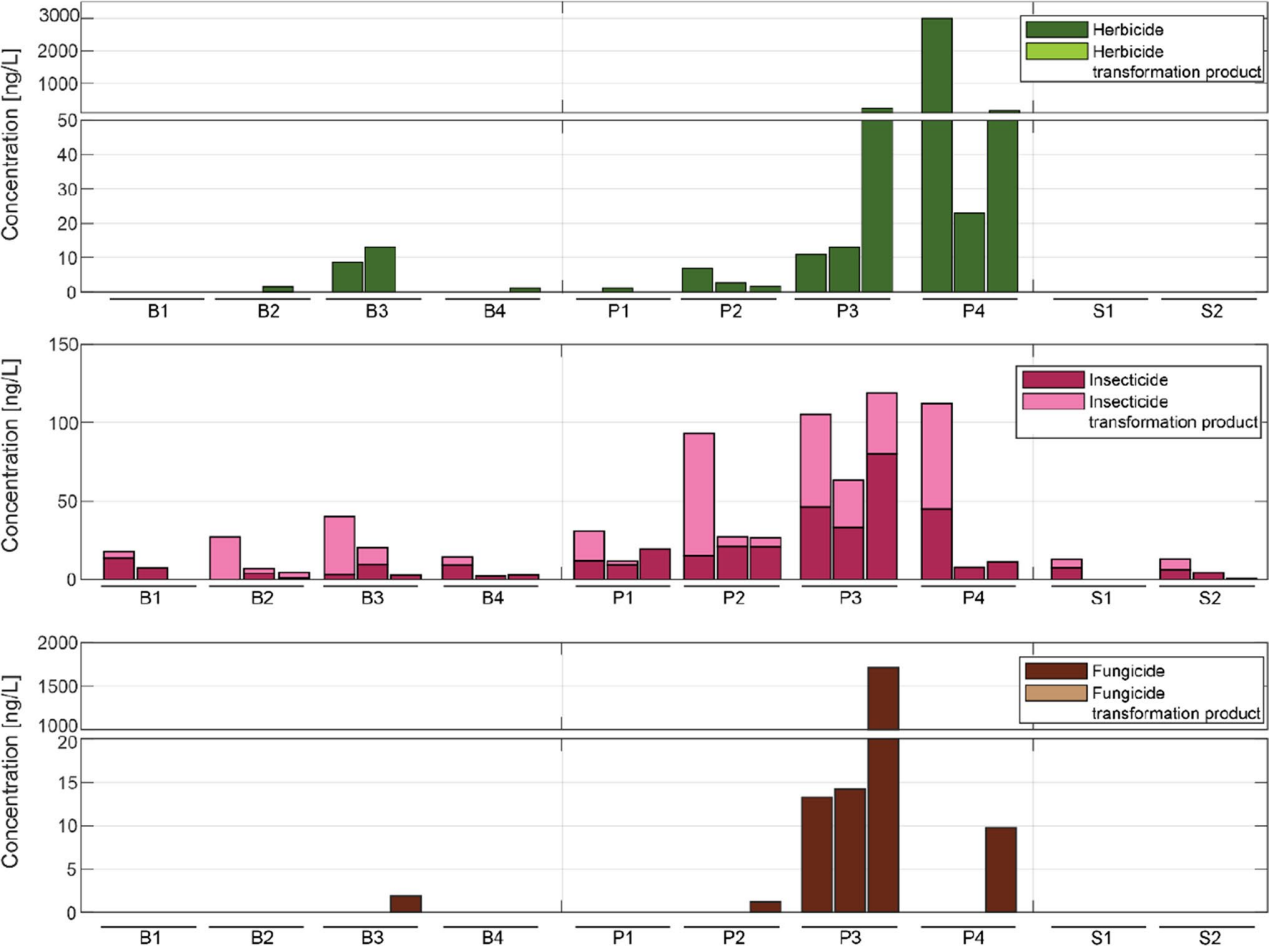
Sum concentration in the drinking water resource through the different sites and times. B1–4: boreholes, P1–4: fetch ponds, S1–2: natural sources

In the drinking water resources, a total of fifteen parent compounds (4 herbicides, 3 fungicides, and 8 insecticides) and two transformation products were detected (Fig. 6 and Supplementary Fig. S6). Additionally, two insect repellents and a biocide (5-Chloro-2-methyl-4-isothiazolin-3-one, CMI) were measured. As for the surface water samples, the concentration distribution was highly skewed. Most concentrations were in the range between 5 and 50 ng/L, with a few very large values exceeding 1000 ng/L (the herbicide 2,4-D and the fungicide carbendazim). Except for these extreme values, the concentration sums were dominated by insecticides and the transformation product of chlorpyrifos 3,4,5-trichloro-2-pyridinol (Supplementary Fig. S6).

Out of nineteen compounds found, only four have guideline values in the WHO guideline (World Health Organization 2017) and only one current pesticide (2,4-D) is regulated in the guideline of potable water (East African Community 2018) (Supplementary Table S8). For none of these compounds, the observed concentrations exceeded guideline values. It is important to note that the guideline for potable water (East African Community 2018) focuses on persistent organochlorine pesticides pollutants (OCPs) such as DDT, lindane, or chlordane. These OCPs were not in our list of target compounds since we decided to focus on currently used, more polar pesticides. However, a non-quantitative screening of PDMS samples taken from a pilot study in Wakiso confirms that the contamination was mainly from current used pesticides (more detail in Supplementary Text S2). Over the 25 OCPs screened, we only detected three compounds above the limit of quantification (> 4 ng/L): endrin, pp’DDD, and pp’DDT. The list of compound is available in Supplementary Table S9.

Not all types of drinking water sources were affected to the same degree (Fig. 6). The overall concentration level in the ponds was the highest and the lowest in the two other sources. The lack of a protective soil layer for the fetch ponds and the absence of effective buffer strips could explain the lower concentration of 2,4-D in the springs than in the wells (equivalent to the boreholes), as suggested by Mekonen et al. (2016). However, one has to consider that there are only two springs (*n* = 6) compared to four boreholes (*n* = 12) and four ponds (*n* = 12).

Many studies suggested that groundwater tends to be more contaminated with transformation products than by the parent pesticides (Dores et al. 2008; Kiefer et al. 2019; Hintze et al. 2020; Mahler et al. 2021), with more limited data specifically on tropical regions (e.g., Karim et al. 2021). As the grab samples were taken from diverse drinking sources, the boreholes and the spring sources should reflect the groundwater contamination, whereas the ponds are most likely contaminated from runoff. Figure 6 illustrates the concentration of the different pesticides groups. Notably, none of the herbicide or fungicide transformation products were detected in the samples. Regarding the two insecticide transformation products, they were both detected in the ponds as well as in the boreholes. The chlorpyrifos transformation product (3,5,6-Trichloro-2-pyridinol (TCP)) occurred in most of the samples from the boreholes (*n* = 10, over 12 samples) and in some of the ponds (*n* = 8, over 12 samples). However, chlorpyrifos was only detected in two boreholes samples and eight from the ponds. Due to high solubility and low octanol/water partition coefficient, TCP has a low sorption capacity on soil (Zhao et al. 2017). This seems to lead to widespread TCP contamination of the aquatic system. On average, there was more TCP in the boreholes or the streams than in the ponds. It could indicate a more substantial contribution from groundwater affecting the quality of drinking water resources.

### Relevance for mitigating pesticide pollution

The observed mismatch between the widespread chlorpyrifos detections and the reported use of the compound as a plant protection product indicates that pesticide exposure to water bodies may not be fully understood if only the use on agriculture crops is considered. This observation suggests that a broader view about pesticide sources and transport pathways is needed to minimize environmental pollution during pesticide use (application and cleaning of material). This conclusion is supported by some recent studies that point toward the relevance of pathways that have been partially overlooked in the past. Ngabirano and Birungi (2022), for example, reported in a recent study on vegetables produced in Uganda that even unsprayed products contain residues. Their main hypothesis was that environmental conditions (high temperature, relative humidity) could cause pesticide volatilization and drift on unsprayed vegetable gardens depending on the pesticide properties. Indeed, a recent paper on currently used pesticides reported pollution of air with atrazine, chlorpyrifos, carbaryl, dimethoate, and malathion in all the samples from Wakiso analyzed (Fuhrimann et al. 2020) from locations outside of treated fields. Studies from tropical regions have demonstrated that volatilization can be a major fate process for compounds such as chlorpyrifos (Dores et al. 2008). Accordingly, it might be advisable to use pesticides with lower volatilization potential.

The relevance of sources and transport pathways strongly depends on the physico-chemical properties of the pesticides used. Many studies in African countries have focused on organochlorine pesticides (e.g., DDT, lindane, endosulfan, and heptachlor) so far. In a systematic review study on pollution in Tanzania, although the prevalent pesticides were OCPs, most of the samples contained pesticides concentration below standard limits (WHO, FAO, US.EPA) (Elibariki and Maguta 2017). However, the data of our research and other recent studies show that other pesticides like chlorpyrifos are also relevant pollutants in Africa (Osafo-Acquaah 1997; Ntow 2001; Elibariki and Maguta 2017; Olisah et al. 2020; Fuhrimann et al. 2020). In Ethiopia, more than half of the water samples showed contamination from malathion, dimethoate, metalaxyl, diazinon, chlorpyrifos, fenitrothion, and endosulfan (Merga et al. 2021). Similarly, Materu et al. (2021) demonstrated the widespread occurrence of polar pesticides in Tanzania.

These findings illustrate that the analytical spectrum has to be widened beyond the OCPs to obtain a full picture of the pesticide pollution levels. As already mentioned by Olisah et al. (2020), the large majority of studies in Africa employed analytical methods which are not the most sensitive. Hence, more collaborating studies such as this one (with international team and local partners) should be promoted to detect a wide range of pesticides and their transformation products. Collaborative monitoring studies would also improve the capacities of the local institutions. Moreover, collaboration increases the potential of building policy decisions by taking into account local situations and prioritized compounds (K’oreje et al. 2020).

### Limitations

In order to gain insight into the pollution with more recent pesticides, we used a method that covered a large number of polar compounds. As a consequence and due to analytical restrictions, our study did not include the classical OCPs. There are also analytical limitations with regard to important pesticides of current use. The herbicide glyphosate and the fungicide mancozeb belonged to the most applied pesticides in the study region (Atuhaire et al. 2017; Staudacher et al. 2020). Their analysis, however, requires special analytical techniques that were not feasible during our study due to a lack of resources. There is a paradox; the most widely used herbicide is also the most difficult to determine (Valle et al. 2019). Therefore, improvement of the analytical methods is encouraged.

We discuss the difference between the results of samples during the same periods taken by different sampling devices. Passive sampling has several advantages, like the easy handling to deploy and its cost. However, some pesticides might not be detected despite their chemical properties. Indeed, the fact that the concentration was measured with time weight average (TWA) concentrations implies that exposure to pesticides during a short time can cause concentration on the passive samplers below the LOQ (Vrana et al. 2007; Schäfer et al. 2008). Options to overcome this issue are more expensive and require more technical devices such as transportable instruments (Stravs et al. 2021). However, we are aware that such instrumentation would not be easily implemented in LMICS. Other reasons such as the different matrix water or the biofilm growing on the PES membrane are factors that can slow the adsorption of the SDBs. There are more uncertainties with membrane-based passive samplers than WLP samplers on the exact quantitative values. With WLP samplers, an internal standard is added before the loading on the SPE cartridges, and the matrix effect is also calculated with the loss effect. For this reason, the WLP samplers might give a better indication of the quantitative value in the rivers. However, passive sampling is recommended for water policy or routine monitoring since the implementation is more affordable (Vrana et al. 2005; Kot-Wasik et al. 2007; Utami et al. 2020).

## Conclusion

Our results demonstrated the occurrence of numerous (polar) pesticides in natural streams and drinking water resources in the study region. Most of these chemicals are currently not included in national water regulations in Uganda and more globally in Africa. For the better protection of the environment but also human health, legislation should be developed further and include pesticides of current use beyond the classical OCPs. These legal updates should be promoted on a global scale, including the tropical countries.

If such regulations were to be implemented, this also required the need for regular monitoring of the drinking sources and the rivers. To that end, passive samplers, as deployed in this study, could be useful tools thanks to their simple handling and deployment and low costs. These low costs can also allow for the use of different sampling systems in parallel. This provides the advantage of creating sampling redundancy minimizing the risk of data gaps and also widening the analytical window. At the same time, the analytical instrumentation and expertise has to be developed in parallel to make sure that the broad pesticide spectrum used by (smallholder) farmers can be adequately captured. The example of chlorpyrifos and its metabolite TCP shows that the list of analytes should also include important transformation products. This compound additionally illustrates the need to not only consider pesticide use for plant protection as the only source of environmental contamination but also consider other applications such as use for vector control or antiparasitics on livestock. Comprehensive knowledge about pesticide use is important for better linking and understanding pesticide use and its impact on environmental health. It is also essential to relate the application of these chemicals with human health outcomes (see, e.g., Joseph et al. 2022.).

## Supplementary Information

Below is the link to the electronic supplementary material.

Supplementary file1 (PDF 1886 KB)

## Data Availability

Upon publication of the article, the data will be made publicly available through the Eawag Research Data Institutional Collection (https://opendata.eawag.ch/).
