# Supplementary material for: Pesticides monitoring in surface water of a subsistence agricultural catchment in Uganda using passive samplers
Source: Environ Sci Pollut Res Int. 2022 Sep 8;30(4):10312–28. doi: 10.1007/s11356-022-22717-2 (PMC9898397; doi:10.1007/s11356-022-22717-2)
Supplement: Supplementary file 1 — Supplementary file1 (PDF 1886 KB) [file 11356_2022_22717_MOESM1_ESM.pdf]

SI: Pesticides monitoring in surface water of a subsistence agricultural catchment in Uganda using passive samplers

Author list:

Christelle Oltramare[1,5], Frederik T. Weiss[1, 2], Philipp Staudacher [1], Oscar Kibirango[3], Aggrey Atuhaire[4], Christian Stamm[1,2]

[1] Eawag: Swiss Federal Institute of Aquatic Science and Technology, 8600 Dübendorf, Switzerland

[2] ETH Zürich, Department of Environmental Systems Science, 8092 Zürich, Switzerland

[3] Directorate of Government Analytical Laboratory (DGAL), Ministry of Internal Affairs, Kampala, Uganda

[4] Uganda National Association of Community and Occupational Health (UNACOH), Kampala, Uganda

Current affiliation:

[5] Center for Primary Care and Public Health (Unisanté), University of Lausanne, 1066 Epalinges-Lausanne, Switzerland

---

Text

Text S1 – Multi-layered cartridge ____ 3

Text S2 – Non-quantitative screening for OCPs ____ 26

Tables

Table S1 - Sampling site location and number of samples per samples type ____ 4

Table S2 - Insecticides analysed in GC-MS/QQQ from the PDMS with ISTD and recoveries. Bold: registered in Uganda. ____ 5

Table S3 - Compounds analysed in LC-MS/MS with ISTD and recoveries. Bold: registered in Uganda. ____ 6

Table S4 - List of synthetic pesticides registered in Uganda and their analytical coverage in this project. The list is an alphabetic extract of pesticides from complete list of all registered agrochemicals in the country, which was combined with the compound list from Tab. S2 and S3. ____ 17

Table S5 - Comparison of occurrence of pesticides with reported used pesticides in Staudacher et al., 2020[1] ____ 18

Table S6 - Table summarizing the Rs calculated (Rs C) and from the literature (Rs L) illustrated in Figure S3 ____ 20

Table S7 – Ecotoxicological water quality standard values ____ 22

Table S8 – Guidelines values for drinking water quality of several pesticides and transformation products from East Africa and WHO compared with the maximum value detected ____ 25

Table S9 - Non-quantitative screening for OCPs ____ 26

Figures

Figure S1 - Illustration of the dynamic in a tributary (Site 2) during one month. Pictures show the response to one day of rain (on the 07.11.207) 16

Figure S2 – Illustration of the sampling rate (Rs) calculated from the onsite samples concentration for each compound detected in the SDB and the WLP. The regression lines represent the relation between the concentration in the water (Cw) and the Mass in the SDB membrane (Mi). The Cw is the concentration detected in WLP samples. The Mi is the mass detected with the SDB disk. The sampling rate is calculated based on the formula: Cw = Mi / (Rs × t) 19

Figure S3 - Correlation of the concentration of Chlorpyrifos detected in in the WLP and PDMS 21

Figure S4 – Chlorpyrifos concentrations from PDMS at the different location. (The sample from week 9-10 at site 5 where lost) 21

Figure S5 - Mixed risk quotient for three-target organism based on the EQS values and the concentrations of pesticides in rives at each respective time and space. 23

Figure S6 – Pesticide concentration from the different drinking sources: boreholes, ponds and springs. 24

Text S1 – Multi-layered cartridge

The multi-layered cartridge were composed in a 6 ml empty cartridge (Supelco, Switzerland) as follow: a frit (Polyethylene, 20 um, Supelco, Switzerland), 200 mg of Supelco-Envicarb, another frit, 350 mg of a mixed phase (100mg Strata-X-AW, Phenomenex, Switzerland: 100 mg Strata-X-CW, Phenomenex, Switzerland; 150 mg Isolute ENV+, Biotage, Switzerland), another frit, 200 mg of OASIS HLB (30 um, Waters, Switzerland) and a last frit.

Table S1 - Sampling site location and number of samples per samples type

Name of sampling site	Source of water sampled	Abbreviation[A]	Coordinates	Year	Sampling period	Sample type	Number of samples
Kaliti	Borehole	B1	00°26’09.51’’ N 32°24’47.28’’ E	2017	Sept-Dec	Grab sample	3
Bumera	Borehole	B2	00°27’28.44’’ N 32°24’47.28’’ E	2017	Sept-Dec	Grab sample	3
Kyabalamba	Borehole	B3	00°27’37.40’’ N 32°23’32.46’’ E	2017	Sept-Dec	Grab sample	3
Bbugga	Borehole	B4	00°27’36.31’’ N 32°27’14.58’’ E	2017	Sept-Dec	Grab sample	3
Kiwazi	Spring well	S1	00°25’37.61’’ N 32°29’04.67’’ E	2017	Sept-Dec	Grab sample	3
Tongolo	Spring well	S2	00°27’09.03’’ N 32°26’40.26’’ E	2017	Sept-Dec	Grab sample	3
Kalenzo	Pond	P1	00°26’31.95’’ N 32°28’44.06’’ E	2017	Sept-Dec	Grab sample	3
Kakunyu	Pond	P2	00°25’13.77’’ N 32°27’51.80’’ E	2017	Sept-Dec	Grab sample	3
Buterega	Pond	P3	00°27’40.75’’ N 32°25’47.58’’ E	2017	Sept-Dec	Grab sample	3
Kongojje	Pond	P4	00°29’06.91’’ N 32°24’59.42’’ E	2017	Sept-Dec	Grab sample	3
Mayanja	River	Site 1	00°24’43.63’’ N 32°29’39.94’’ E	2017	Sept-Dec	WLP [1]	5
						SDB [2]	5
						PDMS [3]	5
Tributary	River	Site 2	00°26’38.73’’ N 32°29’27.23’’ E	2017	Sept-Dec	WLP [1]	5
						SDB [2]	5
						PDMS [3]	5
Tributary	River	Site 3	00°27’04.40’’ N 32°26’33.47’’ E	2017	Sept-Dec	WLP [1]	5
						SDB [2]	5
						PDMS [3]	5
Tributary	River	Site 4	00°27’39.59’’ N 32°23’36.54’’ E	2017	Sept-Dec	WLP [1]	5
						SDB [2]	5
						PDMS [3]	5
Majanya	River	Site 5	00°28’45.37’’ N 32°22’27.60’’ E	2017	Sept-Dec	WLP [1]	5
						SDB [2]	5
						PDMS [3]	5

[A]Abbreviation: B: Borehole, S: Spring, P: Pond
[1] WLP: water-level proportional sampling bottle system
[2] SDB: reverse phase sulfonated styrene-divinylbenzene disks
[3] PDMS: polydimethylsiloxane membranes

Table S2 - Insecticides analysed in GC-MS/QQQ from the PDMS with ISTD and recoveries. Bold: registered in Uganda.

Substance	CAS-No	Internal standard (ISTD)	Optimized analytical parameters	Quality control parameters	
			Quantifier -> Qualifier transition	LOQ (pg/L)	Recovery
Acrinathrin	101007-06-1	Cypermethrin-alpha-d6	542.1 -> 289.0	0.1	93%
Allethrin	584-79-2	Cypermethrin-alpha-d6	303.2 -> 93.0	0.5	105%
**Bifenthrin**	82657-04-3	Bifenthren-d5	181.0 -> 115.0	0.5	115%
**Chlorpyrifos**	2921-88-2	Chlorpyrifos-methyl-d6	349.9 -> 97.0	0.5	76%
Chlorpyrifos-methyl	5598-13-0	Chlorpyrifos-methyl-d6	321.9 -> 289.9	0.2	88%
**Cypermethrin-alpha**	65731-84-2	Cypermethrin-alpha-d6	416.1 -> 127.0	0.1	67%
**Deltamethrin**	52918-63-5	Deltamethrin-d5	504.0 -> 172.0	0.1	100%
Empethrin	54406-48-3	Cypermethrin-alpha-d6	275.2->229.0	0.5	67%
Esfenvalerate	66230-04-4	Esfenvalerate-d7	420.1 -> 125.0	0.1	123%
Etofenprox	80844-07-1	Etofenprox-d5	359.2 -> 189.0	0.1	111%
Fenpropathrin	39515-41-8	Cypermethrin-alpha-d6	350.2 -> 221.0	0.5	24%
Fluvalinate	69409-94-5	Cypermethrin-alpha-d6	503.1 -> 181.0	0.1	86%
Imiprothrin	72963-72-5	Cypermethrin-alpha-d6	319.2 -> 123.0	0.1	79%
**Lambda-Cyhalothrin**	91465-08-6	Cypermethrin-alpha-d6	450.1 -> 141.0	0.1	85%
Phenothrin	26002-80-2	Cypermethrin-alpha-d6	351.2 -> 129.0	0.1	87%
Tefluthrin	79538-32-2	Cypermethrin-alpha-d6	419.1 -> 325.0	0.4	67%
Tetramethrin	7696-12-0	Cypermethrin-alpha-d6	332.2 -> 135.0	0.5	103%

Table S3 - Compounds analysed in LC-MS/MS with ISTD and recoveries. Bold: registered in Uganda.

Substance	CAS-No	Class	Ionization mode	Internal standard (ISTD)	Average LOQ – SDB (not corrected with the sampling rate)	Recovery – SDB [%]	LOQ-WLP	Recovery – WLP [%]
2,4-D	94-75-7	Herbicide	n	2,4-D-D3	0.8	128	1	96
2,4-dimethylphenylformamid	60397-77-5	Insecticide transformation product	p	Oxcarbazepine-d4	3	128	20	122
2,6-Dichlorbenzamid	2008-58-4	Herbicide transformation product	p	2,6-Dichlorbenzamid-3,4,5-D3	2	108	3	84
2-Amino-4-methoxy-6-methyl-1,3,5 triazin	1668-54-8	Herbicide transformation product	p	Chloridazon-methyl-desphenyl-D3	0.2	114	0.4	98
2-Aminobenzimidazol	934-32-7	Fungicide transformation product	p	Sulfathiazol-D4	3	87	0.6	102
2-Imidazolidinethion (=Ethylenethiourea.ETU)	96-45-7	Fungicide transformation product	p	Sotalol-D6	75	62	Na	Na
2-n-Octyl-4-isothiazolin-3-on (OIT)	26530-20-1	Fungicide	p	Octhilinon-D17 (2-n-Octyl-4-isothiazolin-3-on-D17 (OI-D17))	0.3	119	0.5	101
3,5,6-Trichloro-2-pyridinol	6515-38-4	Insecticide transformation product	n	Clofibrin acid-D4	15	92	2	100
3,5-dibromo-4-hydroxybenzoic acid	3337-62-0	Herbicide transformation product	n	Metsulfuron-methyl D3	6	123	7	92
3-Phenoxybenzoe acid	3739-38-6	Insecticide transformation product	n	Diclofenac-D4	0.8	49	5	79
4,5-Dichloro-2-n-octyl-isothiazol-3(2H)-on (DCOIT)	64359-81-5	Wood preservative	p	Fenofibrate-D6	7	74	2	74
4-Isopropylanilin	99-88-7	Herbicide transformation product	p	Fluconazol-D4	5	12	5	74
5-Chloro-2-methyl-4-isothiazolin-3-on (CMI)	26172-55-4	Conservative agent	p	2,6-Dichlorbenzamid-3,4,5-D3	3	108	0.1	18
**Acetamiprid**	160430-64-8	Insecticide	p	Dimethoat-D6	0.4	106	5	104
Acetochlor	34256-82-1	Herbicide	p	Alachlor-D13	3	100	3	117
Acetochlor-ESA	187022-11-3	Herbicide transformation product	n	MCPA-D3	0.4	119	0.1	92
Acetochlor-OXA	194992-44-4	Herbicide transformation product	n	MCPA-D3	0.3	137	3	91
Aclonifen	74070-46-5	Herbicide	p	Indomethacin-D4	20	107	20	21
Alachlor	15972-60-8	Herbicide	p	Alachlor-D13	3	109	2	117
Alachlor-ESA	142363-53-9	Herbicide transformation product	n	MCPA-D3	0.4	119	0.2	86

Alachlor-OXA	171262-17-2	Herbicide transformation product	n	MCPA-D3	3	137	3	100
Aldicarb	116-06-3	Insecticide	p	Venlafaxine-D6	100	0	100	69
Amidosulfuron	120923-37-7	Herbicide	p	Atrazine-D5	2	141	1	101
Asulam	3337-71-1	Herbicide	p	Sulfathiazol-D4	0.3	124	50	67
Atraton (Isobare zu Prometon-Hydroxy)	1610-17-9	Herbicide	p	Primicarb-D6	5	102	0.1	108
**Atrazine**	1912-24-9	Herbicide	p	Atrazine-D5	2	124	2	108
Atrazine-2-Hydroxy	2163-68-0	Herbicide transformation product	p	Atrazine-2-Hydroxy-D5	1	134	2	90
Atrazine-desethyl-2-hydroxy (=Prometon-Hydroxy-Desisopropyl)	19988-24-0	Herbicide transformation product	p	Coffein-D9	0.1	129	Na	Na
Azamethiphos	35575-96-3	Insecticide	p	Simazin-D5	0.3	130	2	74
Azoxystrobin	131860-33-8	Fungicide	p	Azoxystrobin-d4	0.2	119	0.1	105
Azoxystrobin acid	1185255-09-7	Fungicide transformation product	p	Isoproturon-D6p	2	100	4	92
Benalaxyl	98243-83-5	Fungicide	p	Indomethacin-D4	0.3	206	0.1	105
Bentazon	25057-89-0	Herbicide	n	Bentazon-D6	4	123	0.2	117
Benthiavalicarb-isopropyl	177406-68-7	Fungicide	p	Bezafibrat-D4	0.3	143	25	106
Bifenox	42576-02-3	Herbicide	p	Indomethacin-D4	50	142	40	58
Bifenox- acid	53774-07-5	Herbicide transformation product	n	Clofibrin acid-D4	5	88	2	73
Bixafen	581809-46-3	Fungicide	n	Diclofenac-D4	0.1	116	4	95
Boscalid	188425-85-6	Fungicide	p	Methiocarb D3	5	99	10	78
Bromazil	314-40-9	Herbicide	p	Simazin-D5	5	95	7	70
Bromoxynil	1689-84-5	Herbicide	n	Dichlorprop-D6	9	84	0.4	99
Bronopol	52-51-7	Conservative agent	n	Hydrochlorothiazide-13C-D2	3	121	25	131
Butachlor	23184-66-9	Herbicide	p	Fenofibrate-D6	6	62	2	80
Carbendazim	10605-21-7	Fungicide	p	Carbendazim-D4	0.1	130	3	111
Carbetamid	16118-49-3	Herbicide	n	Mesotrion-D3	8	104	55	100
Carbofuran	1563-66-2	Insecticide	p	Metsulfuron-methyl D3	3	86	3	93
Chlorantraniliprole	500008-45-7	Insecticide	p	Methylprednisolol-D3	3	109	4	81

Chlorfenvinphos	470-90-6	Insecticide	p	Propiconazol-D5	4	116	0.8	120
Chloridazon	1698-60-8	Herbicide	p	Chlordiazon-D5	4	106	3	88
Chloridazon-desphenyl	6339-19-1	Herbicide transformation product	p	Chlordiazon-desphenyl-15N2	2	100	6	78
Chloridazon-methyl-desphenyl	17254-80-7	Herbicide transformation product	p	Chloridazon-methyl-desphenyl-D3	2	118	3	98
Chlorothalonil-4-hydroxy	28343-61-5	Fungicide transformation product	n	MCPA-D3	Not detectable	Na	Na	Na
Chlorpyrifos	2921-88-2	Insecticide	p	Chlorpyrifos-D10	2	115	1	105
Chlorpyrifos-methyl	5598-13-0	Insecticide	p	Chlorpyrifos-methyl D6	40	103	10	77
Chlortoluron	15545-48-9	Herbicide	p	Chlortoluron-D6	0.3	129	0.2	104
Clomazon	81777-89-1	Herbicide	p	Methylprednisolol-D3	4	135	3	101
Clothianidin	210880-92-5	Insecticide	p	Clothiandin-D3	8	112	15	98
Cycloxydim	101205-02-1	Herbicide	p	Mefenamin acid-D3	Not detectable	Na	0.1	152
Cyflufenamid	180409-60-3	Fungicide	p	Chlorpyriphos-methyl D6	5	99	3	104
Cymoxanil	57966-95-7	Fungicide	p	N4-Acetyl-Sulfathiazol-D4	5	65	45	53
Cyproconazol	94361-06-5	Fungicide	p	Tebutam-D4	3	102	2	76
Cyprodinil	121552-61-2	Fungicide	p	Cyprodinil D5	0.1	116	25	111
Cyromazin	66215-27-8	Insecticide	p	Morphin-D3	0.1	94	0.4	101
Desethylatrazin	6190-65-4	Herbicide transformation product	p	Desethylatrazin 15N3	0.4	121	3	92
Desisopropylatrazin	1007-28-9	Herbicide transformation product	p	Atrazine-Desisopropyl-D5	0.2	119	3	88
Desmedipham	13684-56-5	Herbicide	p	Methylprednisolol-D3	40	91	125	76
Diazinon	333-41-5	Insecticide	p	Diazinon_D10	0.3	116	0.2	111
Dicamba	1918-00-9	Herbicide	n	Dicamba-D3	20	85	5	62
Dichlorprop	120-36-5	Herbicide	n	Dichlorprop-D6	4	106	2	100
Dichlorvos	62-73-7	Insecticide	p	Simazin-D5	3	123	1	102
Difenoconazol	119446-68-3	Fungicide	p	Chlorpyrifos-methyl D6	100	145	2	98
Diflufenican	83164-33-4	Herbicide	p	Diflufenican-D3	1	119	15	82
Dimefuron	34205-21-5	Herbicide	p	Propazin-D6	3	144	0.2	110

Dimethachlor	50563-36-5	Herbicide	p	Diuron-D6	55	75	3	95
Dimethachlor-ESA		Herbicide transformation product	n	2,4-D-D3	0.5	80	4	79
Dimethachlor-OXA	1086384-49-7	Herbicide transformation product	n	2,4-D-D3	0.7	150	10	79
Dimethenamid	87674-68-8	Herbicide	p	Dimethenamid-D3	2	123	2	105
Dimethenamid-ESA	205939-58-8	Herbicide transformation product	n	Metsulfuron-methyl D3	5	140	4	117
Dimethenamid-OXA	380412-59-9	Herbicide transformation product	n	2,4-D-D3	5	120	4	83
Dimethoate	60-51-5	Insecticide	p	Dimethoat-D6	3	103	3	116
Dinoseb	88-85-7	Herbicide	n	Dichlorprop-D6	0.4	129033	0.2	131
Diuron	330-54-1	Herbicide	p	Diuron-D6	5	108	3	97
Diuron-desdimethyl = 1-(3,4-Dichlorophenyl)urea	2327-02-8	Herbicide transformation product	p	Atrazine-D5	7	54	8	77
Diuron-desmonomethyl (DCPMU) = 1-(3,4-Dichlorophenyl)-3-methylurea	3567-62-2	Herbicide transformation product	p	Diuron-D6	3	116	5	66
DMSA (=N,N-Dimethylaminosulfanilid)	4710-17-2	Fungicide transformation product	p	Carbamazepin-10,11-epoxid-13C, D2	6	124	10	94
Epoxiconazol	133855-98-8	Fungicide	p	Epoxyconazole-D4	0.3	110	0.3	98
Ethephon	16672-87-0	Phytoregulator	n	Hydrochlorothiazide-13C-D2	25	86	Na	Na
Ethofumesat	26225-79-6	Herbicide	p	Dimethenamid-D3	25	100	15	85
Ethofumesat-2-keto	26244-33-7	Herbicide transformation product	p	Carbamazepin-10,11-epoxid-,13C	100	0	35	152
Ethoprophos	13194-48-4	Insecticide	p	Alachlor-D13	0.3	87	0.1	101
Famoxadone	131807-57-3	Fungicide	n	Mefenamin acid-D3	Not detectable	Na	Na	Na
Fenamidone	161326-34-7	Fungicide	p	Dimethenamid-D3	3	119	2	88
Fenhexamid	126833-17-8	Fungicide	p	Octhilinon-D17 (2-n-Octyl-4-isothiazolin-3-on-D17 (OI-D17))	4	96	1	73
Fenoxycarb	79127-80-3	Insecticide	p	Diclofenac-D4	5	103	4	86
Fenpropidin	67306-00-7	Fungicide	p	Diuron-D6	4	72	2	76
Fenpropimorph	67306-03-0	Fungicide	p	Fluoxentine-D5	4	153	5	106
Fipronil	120068-37-3	Insecticide	n	Fipronil-13C2 15N	0.1	50	0.1	78
Fipronil-desulfinyl	205650-65-3	Insecticide transformation product	n	Diclofenac-D4	100	39	1	35

Fipronil-sulfid	120067-83-6	Insecticide transformation product	n	Diclofenac-D4	5	141	3	83
Fipronil-sulfon	120068-36-2	Insecticide transformation product	n	Fipronil-13C2 15N	4	131	3	82
Flonicamid	158062-67-0	Insecticide	p	2,6-Dichlorbenzamid-3,4,5-D3	1	117	9	85
Fluazifop (free acid)	69335-91-7	Herbicide	n	Dichlorprop-D6	5	142	2	118
Fluazinam	79622-59-6	Fungicide	n	Triclosan-D3	3	323	2	222
Fludioxonil	131341-86-1	Fungicide	n	Bicalutamide-D4	7	74	4	77
Flufenacet	142459-58-3	Herbicide	p	Metolachlor-D6	4	110	3	106
Flufenacet-ESA	201668-32-8	Herbicide transformation product	n	Mesotrion-D3	4	118	3	110
Flufenacet-OXA	201668-31-7	Herbicide transformation product	n	MCPA-D3	0.5	97	7	66
Flumioxazin	103361-09-7	Herbicide	p	Methylprednisolol-D3	6	81	Na	Na
Fluopicolide	239110-15-7	Fungicide	p	Valsartan-15N,13C5	4	114	3	104
Fluopyram	658066-35-4	Fungicide	p	Octhilinon-D17 (2-n-Octyl-4-isothiazolin-3-on-D17 (OI-D17))	0.1	116	3	89
Fluoxastrobin	361377-29-9	Fungicide	p	Octhilinon-D17 (2-n-Octyl-4-isothiazolin-3-on-D17 (OI-D17))	3	113	3	85
Fluroxypyr (free acid)	69377-81-7	Herbicide	n	MCPA-D3	15	105	20	70
Flusilazol	85509-19-9	Fungicide	p	Prochloraz D7	0.1	154	3	115
Foramsulfuron	173159-57-4	Herbicide	p	Atomoxetin-D3	2	310	2	119
Fosthiazate	98886-44-3	Insecticide	p	Erythromycin-13C2	3	55	2	78
Haloxyfop	69806-34-4	Herbicide	p	Propiconazol-D5	4	88	2	110
Hexazinon	51235-04-2	Herbicide	p	Simazin-D5	3	156	3	100
Imazamox	114311-32-9	Herbicide	p	Fluconazol-D4	0.5	98	0.9	123
Imidacloprid	138261-41-3	Insecticide	p	Imidacloprid D4	3	115	3	102
Imidacloprid-desnitro	115970-17-7	Insecticide transformation product	p	Chloridazon-methyl-desphenyl-D3	2	106	0.4	95
Imidacloprid-urea	120868-66-8	Insecticide transformation product	p	Imidacloprid D4	3	122	5	94
Iodosulfuron-methyl	144550-36-7	Herbicide	n	MCPA-D3	5	120	2	133
Ioxynil	1689-83-4	Herbicide	n	Metolachlor-ESA D11	5	157	0.2	115
IPBC (=Iodocarb)	55406-53-6	Fungicide	p	N,N-diethyl-3-methylbenzamid-D10 (DEET-D10)	25	48	5	88

Iprodione	36734-19-7	Fungicide	p	Prochloraz D7	9	93	Na	Na
Iprovalicarb	140923-17-7	Fungicide	p	Alachlor-D13	1	102	1	95
Irgarol	28159-98-0	Fungicide	p	Irgarol-D9	6	77	4	121
Irgarol-descyclopropyl	30125-65-6	Fungicide transformation product	p	Sulcotrion-D3	4	149	10	136
Isoproturon	34123-59-6	Herbicide	p	Isoproturon-D6	2	121	0.2	108
Isoproturon-didemethyl = 1-(4-Isoprophenyl)urea	56046-17-4	Herbicide transformation product	p	Carbamazepin-D8	4	99	3	94
Isoproturon-monodemethyl = 1-(4-Isoprophenyl)-3-methylurea	34123-57-4	Herbicide transformation product	p	Carbamazepin-D8	3	150	5	120
Isoxadifen-ethyl	163520-33-0	Herbicide Safener	p	Diclofrnac-D4	4	133	4	100
Isoxaflutole	141112-29-0	Herbicide	p	Simazin-D5	6	74	4	106
Kresoxim-methyl	143390-89-0	Fungicide	p	Diclofenac-D4	7	99	3	111
Lenacil	2164-08-1	Herbicide	p	N,N-diethyl-3-methylbenzamid-D10 (DEET-D10)	3	87	3	92
Linuron	330-55-2	Herbicide	p	Dimethenamid-D3	4	85	4	88
Lufenuron	103055-07-8	Insecticide	n	Triclosan-D3	0.2	305	Na	Na
Maleic hydrazide	123-33-1/10071-13-3	Phytoregulator	p	Sotalol-D6	4	2358	2	53
Mandipropamid	374726-62-2	Fungicide	p	Bezafibrate-D4	4	136	3	89
MCPA	94-74-6	Herbicide	n	MCPA-D3	4	103	3	87
MCPB	94-81-5	Herbicide	n	MCPA-D3	10	55	20	75
Mecoprop	93-65-2	Herbicide	n	Mecoprop-D6	0.4	103	2	91
Mefenpyr-diethyl	135590-91-9	Herbicide Safener	p	Propiconazol-D5	0.3	142	2	131
Mepanipyrim	110235-47-7	Fungicide	p	Valstrsn-15N,13C5	0.1	102	15	88
Mesosulfuron-methyl	74223-64-6	Herbicide	p	Linuron D6	2	208	4	93
Mesotrion	104206-82-8	Herbicide	p	Mesotrion-D3	6	108	7	84
Mesotrion-MNBA	110964-79-9	Herbicide transformation product	n	Mesotrion-D3	7	156	79	130
Metalaxyl	57837-19-1	Fungicide	p	Metalaxy-D6	2	121	2	111
Metaldehyde	9002-91-9	Mulloscicide	n	Mesotrion-D3	Not detectable	Na	Na	Na
Metamitron	41394-05-2	Herbicide	p	Dimethoat-D6	5	85	Na	Na

Metamitron-desamino	36993-94-9	Herbicide transformation product	p	Metoprolol-D7	4	133	4	106
Metazachlor	67129-08-2	Herbicide	p	Atrayin-D5	2	148	6	117
Metazachlor-ESA	172960-62-2	Herbicide transformation product	n	2,4-D-D3	4	142	5	77
Metazachlor-OXA	1231244-60-2	Herbicide transformation product	p	Desethylatrazin 15N3	3	135	4	75
Metconazole	125116-23-6	Fungicide	p	Chlorpyrifos-methyl D6	3	178	2	92
Methidathion	950-37-8	Insecticide	p	Irgarol-D9	10	103	9	98
Methiocarb	2032-65-7	Insecticide	p	Methiocarb D3	5	99	0.3	103
Methiocarb-sulfoxide	2635-10-1	Insecticide transformation product	p	Dimethoat-D6	3	96	Na	Na
Methomyl	16752-77-5	Insecticide	p	Carbendazim-D4	30	131	7	119
Methoxyfenozid	161050-58-4	Insecticide	p	Valsartan-15N,13C5	3	130	4	76
Metolachlor	51218-45-2	Herbicide	p	Metolachlor-D6	0.1	121	1	102
Metolachlor-ESA	171118-09-5	Herbicide transformation product	n	Metolachlor-ESA D11	0.3	122	0.3	92
Metolachlor-Morpholinon	120375-14-6	Herbicide transformation product	p	Atrazine-D5	0.1	110	0.1	118
Metolachlor-OXA	152019-73-3	Herbicide transformation product	n	Metolachlor-ESA D11	4	121	3	102
Metosulam	139528-85-1	Herbicide	p	Verapamil-D6	3	168	3	122
Metoxuron	19937-59-8	Herbicide	p	Venlafaxine-D6	4	1	3	96
Metrafenone	220899-03-6	Fungicide	p	Chlorpyrifos-methyl D6	0.4	189	3	112
Metribuzin	21087-64-9	Herbicide	p	Simazin-D5	Not detectable	Na	3	92
Metribuzin-Desamino (DA)	35045-02-4	Herbicide transformation product	p	Carbamazepin-D8	3	259	4	94
Metsulfuron-methyl	74223-64-6	Herbicide	p	Metsulfuron-methyl D3	0.2	102	2	92
Monocrotophos	6923-22-4	Insecticide	p	N4-Acetyl-Sulfathiazol-D4	3	184	2	146
Monolinuron	1746-81-2	Herbicide	p	Carbamazepin-D8	4	91	4	89
Monuron	150-68-5	Herbicide	p	Propranolol-D7	4	277	4	196
Myclobutanil	88671-89-0	Fungicide	p	Bezafibrat-D4	0.3	137	0.2	95
N-(2,4-dimethylphenyl)-N-methylformamidin	33089-74-6	Insecticide transformation product	p	N4-Acetyl-Sulfathiazol-D4	3	112	3	83
N,N-diethyl-3-methylbenzamid (DEET)	134-62-3	Insect repellent	p	N,N-diethyl-3-methylbenzamid-D10 (DEET-D10)	1	122	50	189

N,N-dimethyl-N'-(4-methylphenyl)-sulfamid	66840-71-9	Wood preservative	p	Carbamazepin-D8	6	74	5	69
Napropamid	15299-99-7	Herbicide	p	Alachlor-D13	3	98	2	97
Nicosulfuron	111991-09-4	Herbicide	p	Simazin-D5	2	182	2	117
Orbencarb	34622-58-7	Herbicide	p	Propiconazol-D5	4	131	5	109
Oryzalin	19044-88-3	Herbicide	p	Prochloraz D7	7	101	21	79
Oxamyl	23135-22-0	Insecticide	n	2,4-D-D3	100	104	Na	Na
Oxasulfuron	144651-06-9	Herbicide	p	Oxcarbazepine-D4	2	159	2	148
Oxyfluorfen	42874-03-3	Herbicide	p	Propiconazol-D5	4	92	0.2	83
Penconazol	66246-88-6	Fungicide	p	Diclofenac-D4	3	143	2	117
Pencycuron	66063-05-6	Fungicide	p	Diflufenican-D3	5	162	2	110
Pethoxamid	106700-29-2	Herbicide	p	Alachlor-D13	0.1	101	2	102
Picaridin (Icaridin)	119515-38-7	Insect repellent	p	Dimethenamid-D3	0.8	128	0.9	109
Pirimicarb	23103-98-2	Insecticide	p	Pirimicarb D6	0.3	119	2	112
Prochloraz	67747-09-5	Fungicide	p	Prochloraz D7	1	122	0.3	97
Procymidone	32809-16-8	Fungicide	p	Octhilinon-D17 (2-n-Octyl-4-isothiazolin-3-on-D17 (OI-D17))	100	73	Na	33
Profenophos	41198-08-7	Insecticide	p	Diflufenican-D3	0.5	79	2	77
Prometon	1610-18-0	Herbicide	p	Oxcarbazepine-D4	0.1	113	0.2	106
Prometryn + Terbutryn	7287-19-6	Herbicide	p	Terbutryn-D5	0.1	126	20	90
Propachlor	1918-16-7	Herbicide	p	Isoproturon-D6	3	129	2	103
Propachlor-ESA	123732-85-4	Herbicide transformation product	n	2,4-D-D3	3	152	4	83
Propachlor-OXA	70628-36-3	Herbicide transformation product	n	2,4-D-D3	20	123	20	112
Propamocarb	24579-73-5	Fungicide	p	Codein-13C,D3	0.2	133	3	76
Propanil	709-98-8	Herbicide	p	Bezafibrat-D4	5	93	4	74
Propaquizafop	111479-05-1	Herbicide	p	Fenofibrate-D6	0.7	92	2	88
Propazine-2-hydroxy (=Prometon-Hydroxy)	7374-53-0	Herbicide transformation product	p	Fluconazol-D4	0.5	132	0.2	113
Propiconazol	60207-90-1	Fungicide	p	Propiconazol-D5	4	126	3	106

Propyzamide	23950-58-5	Herbicide	p	Valsartan-15N,13C5	4	114	3	96
Prosulfocarb	52888-80-9	Herbicide	p	Diflufenican-D3	5	84	2	91
Prosulfuron	94125-34-5	Herbicide	p	Bezafibrat-D4	3	153	2	109
Prothioconazole-desethio	120983-64-4	Fungicide transformation product	p	Prochloraz D7	3	180	3	109
Prothiophos	34643-46-4	Insecticide	n	Na	Not detectable	Na	Na	Na
Pymetrozin	123312-89-0	Insecticide	p	Atenolol-D7	2	125	3	153
Pyraclostrobin	175013-18-0	Fungicide	p	Propiconazol-D5	3	119	3	120
Pyridat	55512-33-9	Herbicide	p	Indomethacin-D4	1	438	Na	Na
Pyrimethanil	53112-28-0	Fungicide	p	Verapamil-D6	1	203	2	111
Pyrimidinol	2814-20-2	Insecticide transformation product	p	Carbendazim-D4	15	120	55	82
Pyroxsulam	422556-08-9	Herbicide	n	Metsulfuron-methyl D3	7	104	2	100
Quinoclamine	2797-51-5	Herbicide	p	Oxcarbazepine-D4	5	102	9	61
Rimsulfuron	122931-48-0	Herbicide	p	Carbamazepin-D8	2	102	8	247
Simazin	122-34-9	Herbicide	p	Simazin-D5	0.1	113	2	112
Simazin-2-hydroxy	2599-11-3	Herbicide transformation product	p	Sulfathiazol-D4	0.3	104	0.4	129
Simeton	673-04-1	Herbicide	p	Clothianidin-D3	0.4	106	0.1	119
Spirotetramat	203313-25-1	Insecticide	p	Octhilinon-D17 (2-n-Octyl-4-isothiazolin-3-on-D17 (OI-D17))	0.2	113	3	201
Spiroxamin	118134-30-8	Fungicide	p	Linuron D6	0.1	96	2	82
Sulcotrion	99105-77-8	Herbicide	p	Sulcotrion-D3	4	113	5	88
Sulcotrion-CMBA	53250-83-2	Herbicide transformation product	n	Hydrochlorothiazide-13C-D2	3	117	43	59
Sulfentrazon	122836-35-5	Herbicide	n	Metolachlor-ESA D11	5	106	5	75
Sulfosulfuron	141776-32-1	Herbicide	p	Linuron D6	2	194	3	83
Tebuconazol	107534-96-3	Fungicide	p	Diazinon_D10	0.3	113	2	5
Tebufenozid	112410-23-8	Insecticide	p	Prochloraz D7	3	180	4	123
Tebutam	35256-85-0	Herbicide	p	Tebutam-D4	0.1	117	2	108
Teflubenzuron	83121-18-0	Insecticide	p	Indomethacin-D4	6	98	3	64

Tepraloxydim	149979-41-9	Herbicide	p	Tebutam-D4	4	92	2	85
Terbacil	5902-51-2	Herbicide	n	Bicalutamide-D4	9	61	8	61
Terbumeton	33693-04-8	Herbicide	p	Simazin-D5	0.1	103	0.1	90
Terbutryn + Prometryn	886-50-0	Herbicide	p	Terbutryn-D5	0.1	124	0.2	91
Terbutylazin	5915-41-3	Herbicide	p	Terbutylazin-D5	3	125	2	103
Terbutylazin-2-hydroxy	66753-07-9	Herbicide transformation product	p	Fluconazol-D4	5	132	2	104
Terbutylazin-desethyl	30125-63-4	Herbicide transformation product	p	Carbamazepin-D8	0.4	98	3	97
Thiabendazol	148-79-8	Fungicide	p	Coffein-D9	0.1	90	3	100
Thiacloprid	111988-49-9	Insecticide	p	5-methyl-1H- Benzotriazol	5	104	6	77
Thiacloprid-amide	676228-91-4	Insecticide transformation product	p	Atrazine-2-Hydroxy-D5	3	127	5	72
Thiamethoxam	153719-23-4	Insecticide	p	Thiamethoxame D3	2	114	3	92
Thiencarbazone	317815-83-1	Herbicide	n	Sulcotrion-D3	8	73	2	121
Thifensulfuron-methyl	79277-27-3	Herbicide	n	Metsulfuron-methyl D3	0.7	120	20	97
Triazophos	24017-47-8	Insecticide	p	Valsartan-15N,13C5	3	156	2	95
Triazoxide	72459-58-6	Fungicide	p	Oxcarbazepine-D4	3	143	3	93
Tribenuron-methyl	101200-48-0	Herbicide	p	Atrazine-D5	100	12	Na	Na
Triclocarban	101-20-2	Conservative agent	p	Diflufenican-D3	6	90	1	66
Triclopyr	55335-06-3	Herbicide	p	Clofibrin acid-D4	Not detectable	Na	Na	Na
Triclosan	3380-34-5	Desinfection agent	n	Triclosan-D3	1	115	3	94
Trifloxystrobin	141517-21-7	Fungicide	p	Mefenamin acid-D3	0.5	149	5	123
Triflusulfuron-methyl	126535-15-7	Herbicide	p	Methiocarb D3	2	193	4	107
Trinexapac-ethyl	95266-40-3	Herbicide	p	Diuron-D6	3	130	3	108
Tritosulfuron	142469-14-5	Herbicide	p	Bezafibrat-D4	5	95	2	103

Figure S1 - Illustration of the dynamic in a tributary (Site 2) during one month. Pictures show the response to one day of rain (on the 07.11.207)

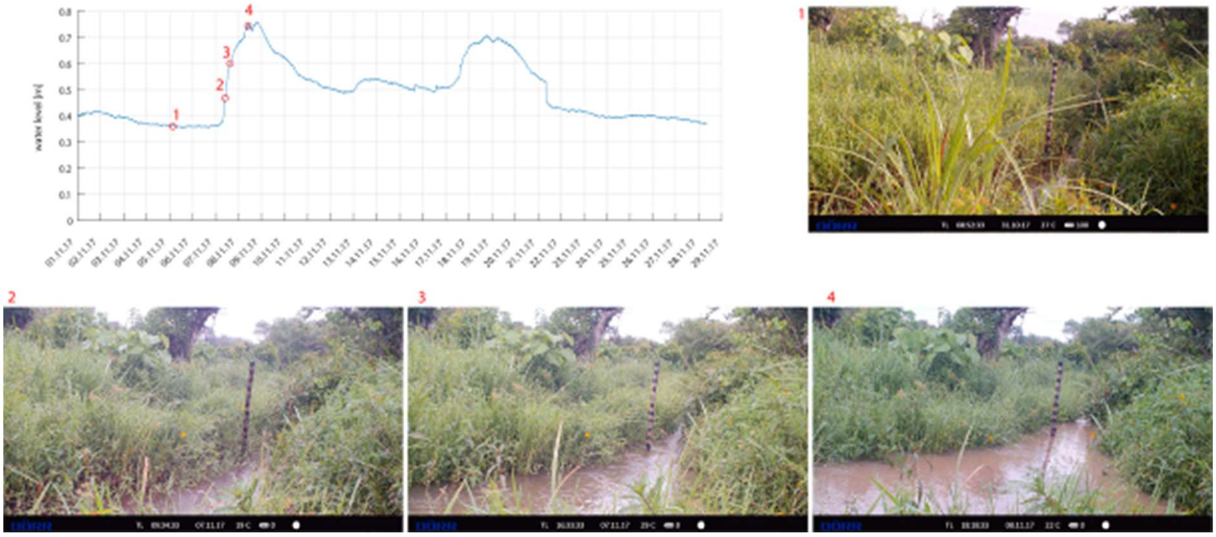

Table S4 - List of synthetic pesticides registered in Uganda and their analytical coverage in this project. The list is an alphabetic extract of pesticides from complete list of all registered agrochemicals in the country, which was combined with the compound list from Tab. S2 and S3.

Registered compound	GC-MS	LC-MS
2,4 D amine		1
2,4-D ethylhexyl		
Acetamiprid		
Alpha-Cypermethrin	1	
Ametryn		
Atrazine		1
Azadirachtin		
Azoxystrobin		1
Bentazone		1
Beta- Cypermethrin		
Betacyfluthrin		
Bifenazate		
Bifenthrin	1	
Bispyribac		
Bixafen		1
Brodifacoum		
Bromoxynil		
Butachlor		1
Carbendazim		1
Carbofuran		1
Carbosulfan		
Carfentrazone ethyl		
Chlorimuron-ethyl		
Chloroacetamide		
Chlorothalonil		
Chlorpropham		
Chlorpyrifos	1	1
Chlorpyrifos ethyl		
Clethodim		
Clomazone		1
Cyhalofop butyl		
Cymoxanil		1
Cypermethrin	1	
Cyproconazole		1
Deltamethrin	1	
Dicamba		1
Dichlorvos		1
Difenoconazole		1
Diflubenzuron		
Dimethenamid-P		1
Dimethoate		1
Dimethomorph		
Diuron		1
Epoxiconazole		1
Ethoxysulfuron		
Fenitrothion		
Fenoxaprop-P-ethyl		
Fenvalerate		
Fipronil		1
Fluazifop-P-butyl		
Flubendiamide		
Fludioxonil		1
Fluopicolide		1
Fluopyram		1
Fomesafen		
Glufosinate Ammonium		
Glyphosate		
Hexaconazole		

Registered compound (cont.)	GC-MS	LC-MS
Imazamox		1
Imazapyr		
Imazethapyr		
Imidacloprid		1
Indaziflam		
Indoxacarb		
Isoxadifen-ethyl		1
Lambdacyhalothrin	1	
Lufenuron		1
Malathion		
Mancozeb		
Maneb		
Mecoprop-P		1
Mefenpyr- diethyl		1
Mesotrione		1
Metalaxyl		1
Metalaxyl-M		
Metolachlor		1
Metribuzin		1
Nicosulfron		1
Octanoate		
Oxadiazon		
Oxyfluorfen		1
Pendimethalin		
Penoxsulam		
Penoxsulam		
Permethrin		
Pirimiphos Methyl		
Profenofos		
Propamocarb hydrochloride		
Propanil		
Propiconazole		
Propineb		
Pyraclostrobin		1
Quinclorac		
Quizalofop-p-ethyl		
Saflufenacil		
S-Metolachlor		1
Spirodiclofen		
Spirotetramat		1
Sulfentrazone		1
Sulfoxaflor		
Tebuconazole		1
Tembotrione		
Terbuthylazine		1
Thiamethoxam		1
Thiobencarb		
Thiophanate methyl		
Topramezone		
Triafamone		
Triazophos		1
Trichlopyr butoxyethyl ester		
Tricyclazole		
Trifloxystrobin		1
Zeta Cypermethrin		
**Total coverage**	**6**	**44**

Table S5 - Comparison of occurrence of pesticides with reported used pesticides in Staudacher et al., 2020[1]

	Share of pesticide users over the last 12 months [Staudacher et al., 2020][1]	Occurrence of pesticide detected [2,3]	
	[%]	in river (n=25) [%]	in drinking source (n=30) [%]
2,4-D	33	76	43
Acetamiprid	no data obtained	Nd	23
Atrazine	no data obtained	76	17
Azoxystrobin	no data obtained	28	17
Benalaxyl	no data obtained	Nd	10
Bentazon	no data obtained	40	Nd
Carbendazim	no data obtained	44	43
Carbaryl	4	Nd	Nd
Carbofuran	3	Nd	10
Chlorfenvinphos	no data obtained	Nd	13
Chlorpyrifos	6	96	33
Chlortoluron	no data obtained	Nd	10
Cypermethrin	42	48	Nd
Deltamethrin	0	36	Nd
Diazinon	3	Nd	20
Dichlorvos	11	16	70
Dimethoate	9	Nd	7
Etofenprox	no data obtained	8	Nd
Ethoprophos	no data obtained	Nd	20
Fluvalinate-tau	no data obtained	4	Nd
Glyphosate[3]	55	Na	Na
Imidacloprid	no data obtained	28	Nd
Isoproturon	no data obtained	44	Nd
Lamdba-cyhalothrin	10	8	Nd
Metalaxyl	no data obtained	80	17
Mancozeb[3]	38	Na	Na
Paraquat	3	Na	Na
Permethrin	2	Na	Na
Phenothrin	no data obtained	28	Nd
Picaridin	no data obtained	92	97
Profenofos	34	Nd	27
Prometon	no data obtained	Nd	20
Pyrimidinol	no data obtained	72	Nd
Thiamethoxam	no data obtained	16	Nd

[1]: Staudacher, P., Fuhrimann, S., Farnham, A., Mora, A.M., Atuhaire, A., Niwagaba, C., Stamm, C., Eggen, R.I., Winkler, M.S., 2020. Comparative Analysis of Pesticide Use Determinants Among Smallholder Farmers From Costa Rica and Uganda. Environ. Health Insights 14, 117863022097241. https://doi.org/10.1177/1178630220972417

[2] : Nd: not detected.

[3] Na : not analysed. Glyphosate, Mancozeb, Paraquat and Permethrin are highlighted in grey since they are not analysed in the current study.

Figure S2 – Illustration of the sampling rate (Rs) calculated from the onsite samples concentration for each compound detected in the SDB and the WLP. The regression lines represent the relation between the concentration in the water (Cw) and the Mass in the SDB membrane (Mi). The Cw is the concentration detected in WLP samples. The Mi is the mass detected with the SDB disk. The sampling rate is calculated based on the formula: Cw = Mi / (Rs × t)

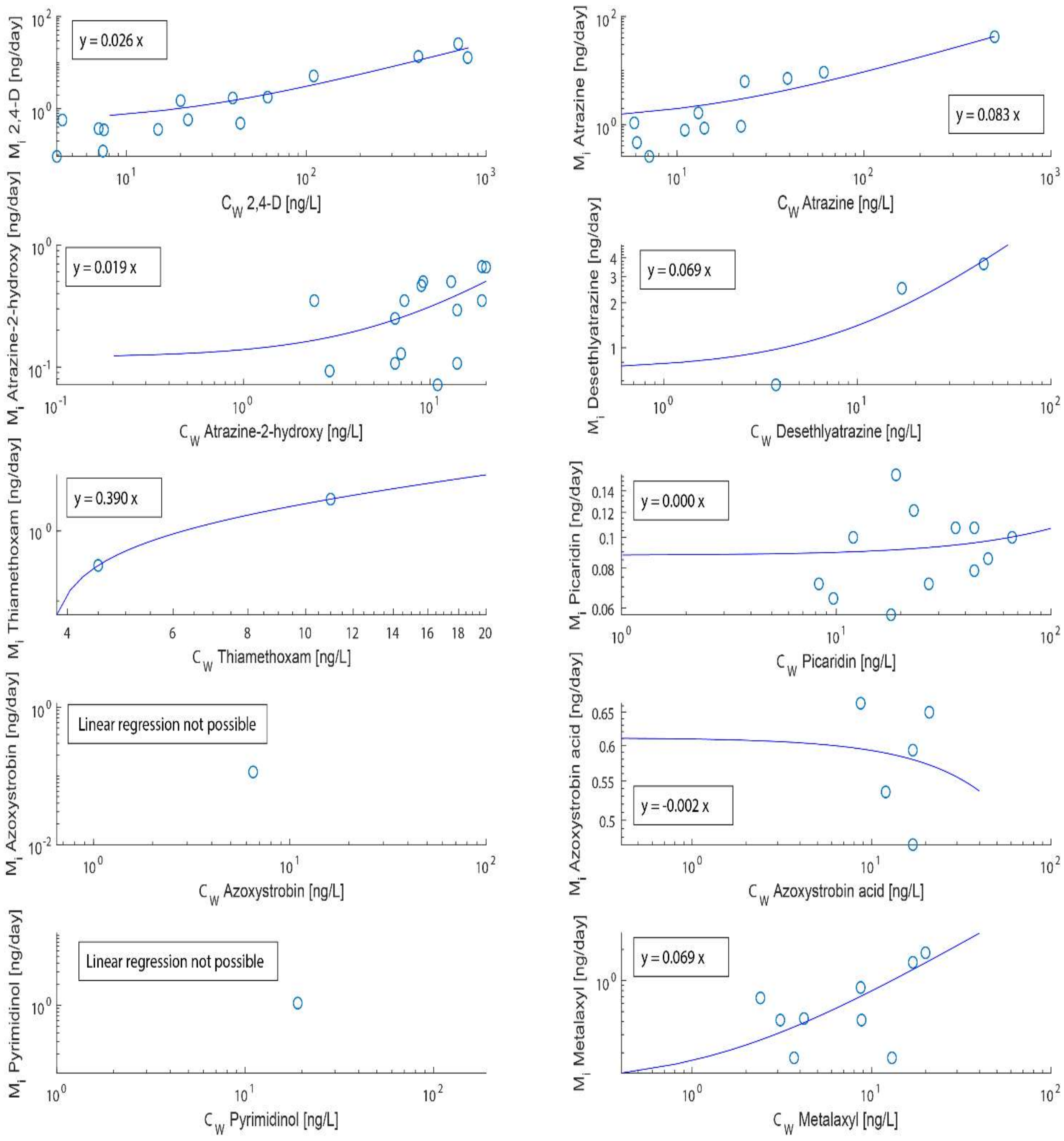

Table S6 - Table summarizing the Rs calculated (Rs C) and from the literature (Rs L) illustrated in Figure S3

	Rs C [L/d]	Rs L [L/d] [1]	Ratio	WLP results [min;median;max]	SDB results [min;median;max]	Fold difference
2,4-D	0.026	0.02	1.30	4.1 ;22 ;790	1.3 ;8.1;360	1.30
Atrazine	0.086	0.03	2.87	0.8 ;6.1 ;500	0.7 ;6.5 ;590	2.87
Atrazine-2-hydroxy	0.028	0.03	0.93	0.8 ;7.15 ;21	0.1 ;3.5 ;9.3	1.07
Desethylatrazine	0.090	0.15	0.60	3.8 ;17 ;45	2.2 ;7.9 ;51	1.67
Thiamethoxam	0.233	0.06	3.88	4.5 ;7.75 ;11	2.1 ;14.25 ;40	3.88
Picaridin	0.002	0.01	0.20	4.7 ;19 ;66	0.5 ;1.1 ;2.2	5.00
Azoxystrobin	ND	0.09		0.6 ;2.9 ;6.5	0.4 ;0.75 ;1.6	NA
Azoxystrobin acid	0.035	0.07	0.50	3.6 ;12 ;21	1.1 ;1.45 ;9.3	2.00
Pyrimidinol	ND	0.01		0.05 ;15 ;67	3.1 ;9.95 ;32	NA
Metalaxyl	0.077	0.06	1.28	2.4 ;6.45 ;20	1.2 ;3.1 ;26	1.28

[1]: Ahrens, L., Daneshvar, A., Lau, A.E., Kreuger, J., 2015. Characterization of five passive sampling devices for monitoring of pesticides in water. J. Chromatogr. A 1405, 1–11. https://doi.org/10.1016/j.chroma.2015.05.044

Figure S3 - Correlation of the concentration of Chlorpyrifos detected in in the WLP and PDMS

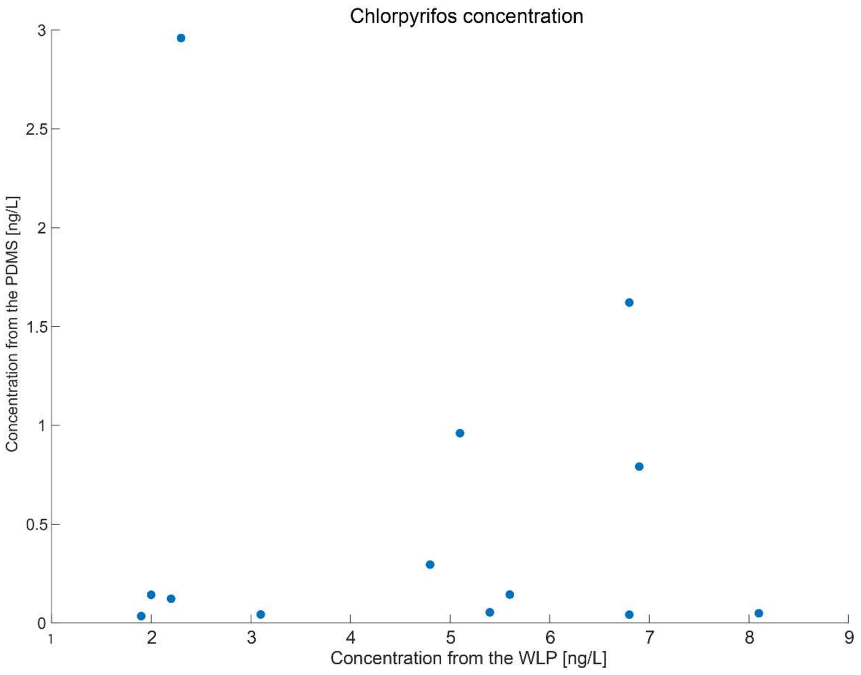

Figure S4 – Chlorpyrifos concentrations from PDMS at the different location. (The sample from week 9-10 at site 5 where lost)

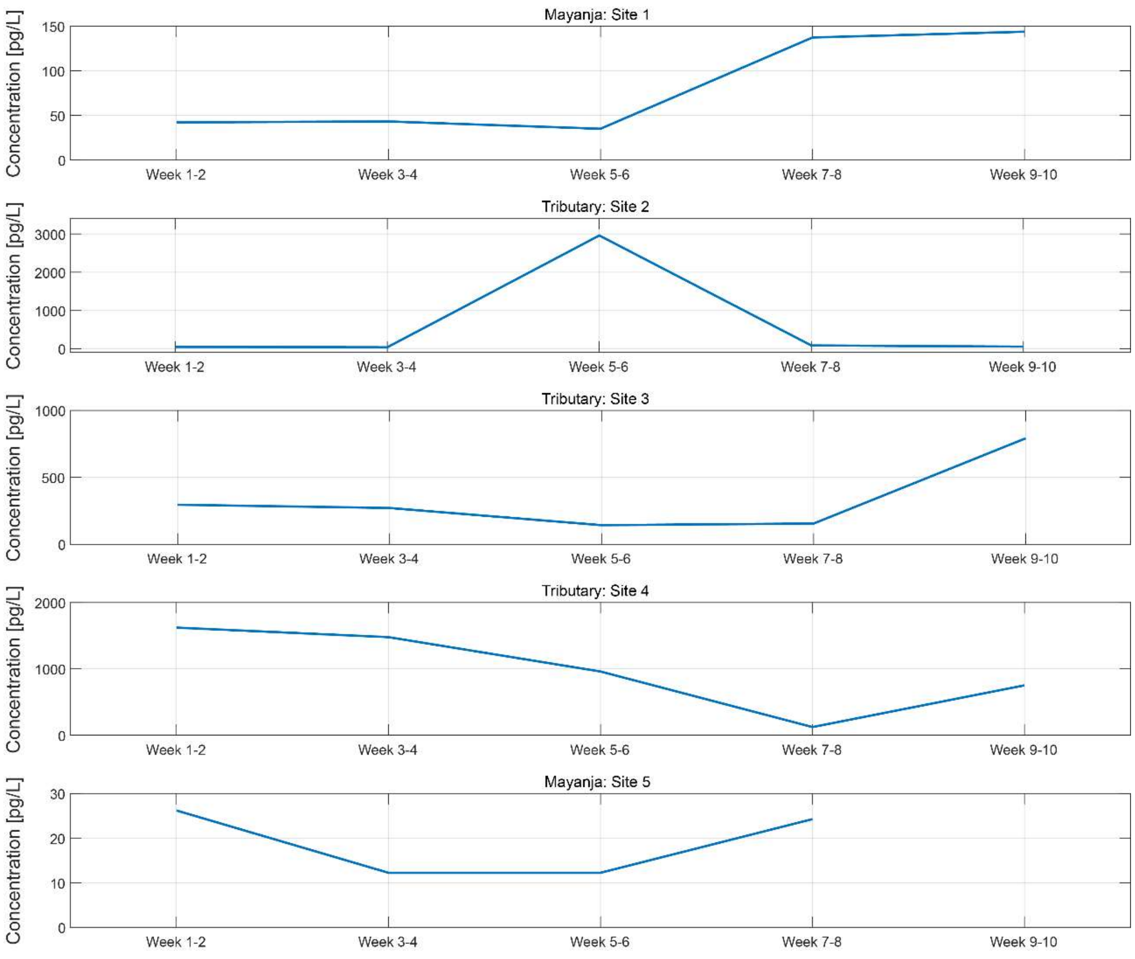

Table S7 – Ecotoxicological water quality standard values

Compound detected at the river sites	AA-EQS[1] [ng/L]	Max Value detected [ng/L] (WLP / SDB)
2,4-D	600	790 / 360
3,5,6-Trichloro-2-pyridinol		18 / 1
5-Chloro-2-methyl-4-isothiazolin-3-on (CMI)		110 / -
Atrazine	600	500 / 590
Atrazine-2-hydroxy		21 / 9
Azoxystrobin	200	6.5 / 2
Azoxystrobin acid		21 / 9
Bentazon	270000	8 / 2
Carbendazim	440	34 / -
Chlorpyrifos	0.46	8 / -
Cypermethrin	0.03	
Deltamethrin	0.0017	
Desethlyatrazine		45 / 51
Desisopylatrazine		14 / 12
Dichlorvos		7.3 / -
Etofenprox		
Fluvalinate		
Imidacloprid	13	- / 6
Isoproturon	640	4 / 5
Lambda cyhalothrin	0.022	
Metalaxyl	20000	20 / 26
Phenonthrin		
Picaridin (Icaridin)		66 / 2
Propazine-2-hydroxy		1 / -
Pyrimidinol		67 / 32
Thiamethoxam	42	11 40

[1] AA-EQS: Annual average Environmental Quality Standards, Datasouce: Ecotoxcenter, EAWAG/EPFL, 2020. Proposals for Acute and Chronic Quality Standards, Oekotoxzentrum. https://www.ecotoxcentre.ch/ and European Commission, 2018. Technical Guidance for Deriving Environmental Quality Standards Guidance Document No. 2

Figure S5 - Mixed risk quotient for three-target organism based on the EQS values and the concentrations of pesticides in rives at each respective time and space.

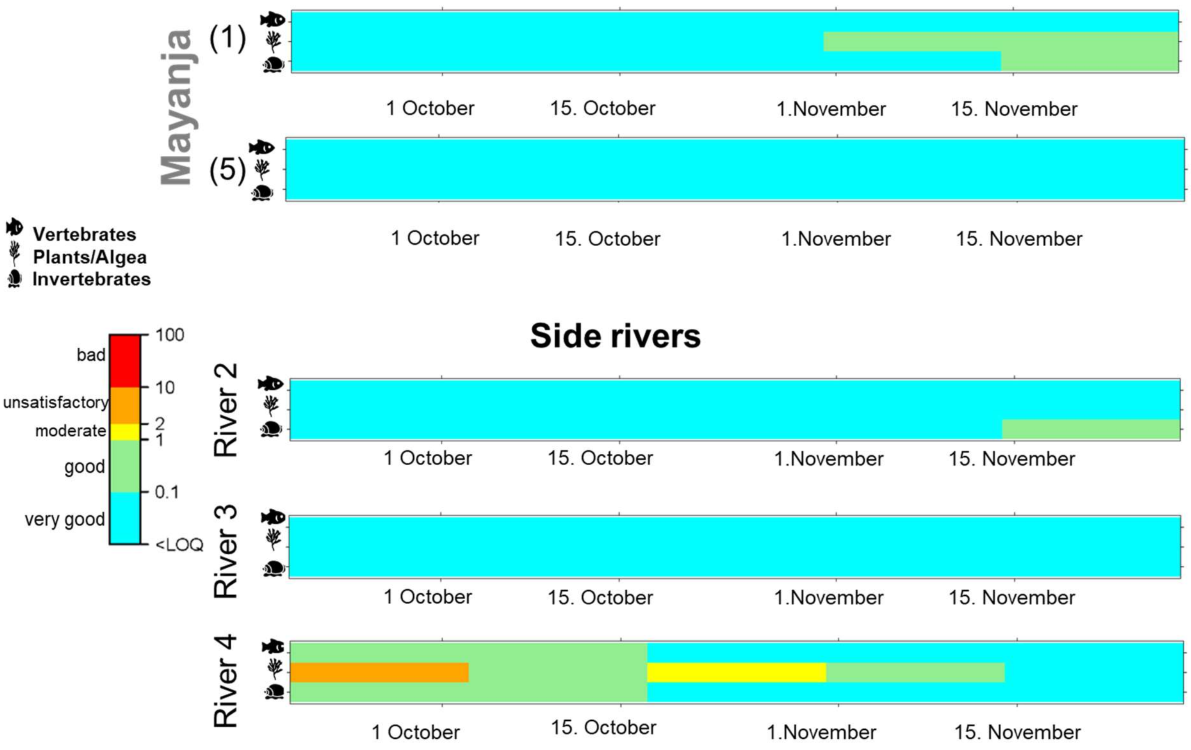

Figure S6 – Pesticide concentration from the different drinking sources: boreholes, ponds and springs.

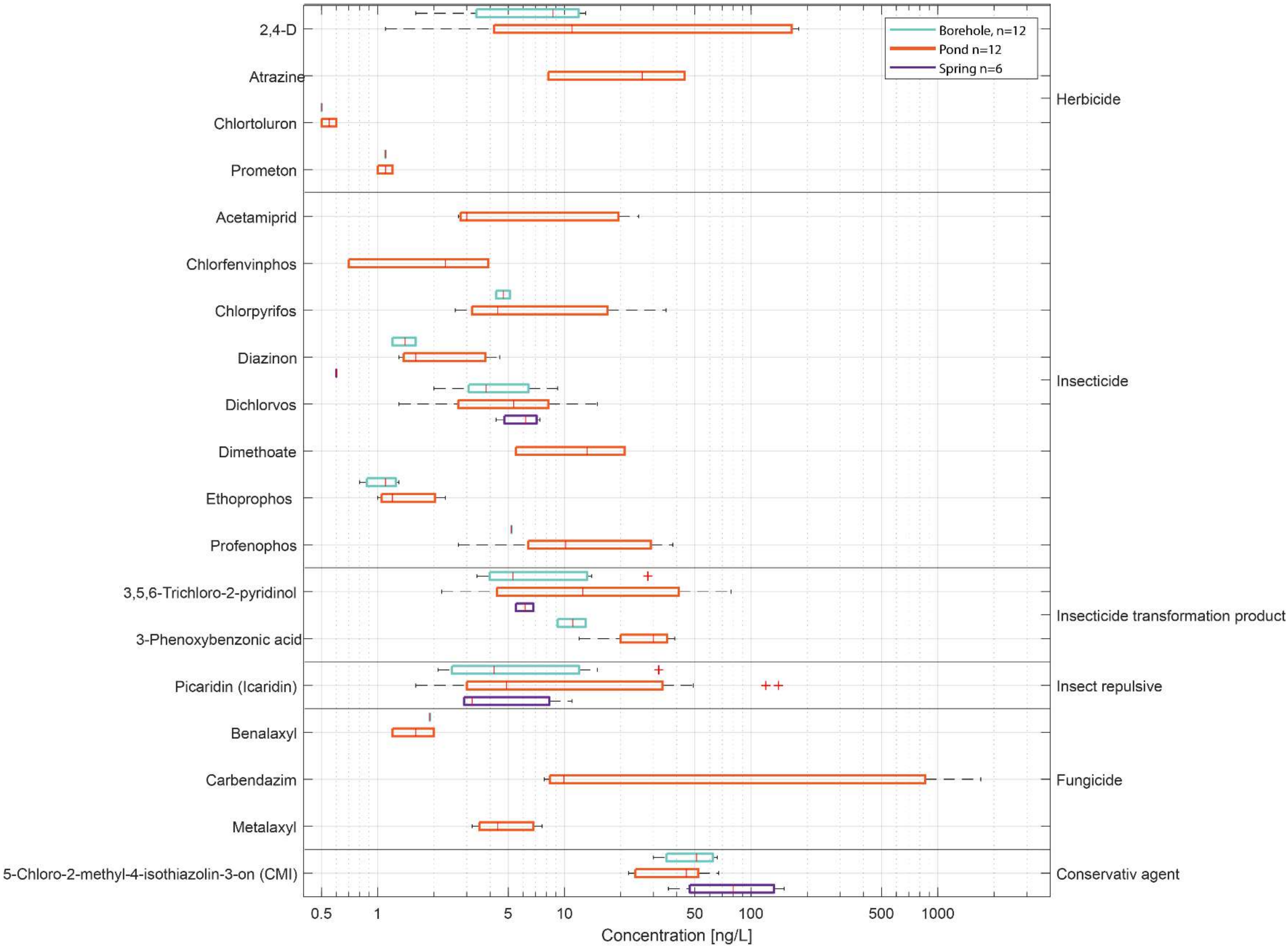

Table S8 – Guidelines values for drinking water quality of several pesticides and transformation products from East Africa and WHO compared with the maximum value detected

Compound detected in the drinking source	Guidelines for drinking water quality East Africa 2018 [µg/L] [1]	Guidelines for drinking water quality WHO 2017 [µg/L] [2]	Max Value detected [µg/L]
2,4-D	30	30	3
3,5,6-Trichloro-2-pyridinol	Na	Na	0.078
3-Phenoxybenzoe acid	Na	Na	0.039
5-Chloro-2-methyl-4-isothiazolin-3-on (CMI)	Na	Na	0.15
Acetamiprid	Na	Na	0.025
Atrazine	Na	100	0.044
Benalaxyl	Na	Na	0.002
Carbendazim	Na	Na	1.7
Chlorfenvinphos	Na	Na	0.0039
Chlorpyrifos	Na	30	0.035
Chlortoluron	Na	Na	0.0006
Diazinon	Na	Unlikely to occur in drinking-water	0.0045
Dichlorvos	Na	Occurs in drinking-water or drinking-water sources at concentrations well below those of health concern	0.015
Dimethoate	Na	6	0.021
Ethoprophos	Na	Na	0.0023
Metalaxyl	Na	Na	0.0076
Picaridin (Icaridin)	Na	Na	0.14
Profenophos	Na	Na	0.038
Prometon	Na	Na	0.0012

[1] : East African Community, 2018. FINAL DRAFT EAST AFRICAN STANDARD.

[2] : World Health Organization, 2017. Guidelines for drinking-water quality ISBN: 978-92-4-154995-0

Text S2 – Non-quantitative screening for OCPs

We organized a pilot study in order to optimize the sampling method and the site location. We used the extract form PDMS samples to check the contamination of OCPs in the surface water in Wakiso, Uganda. The PDMS used for this screening were deployed for 14 days in March 2017 at site location 2 and 3. After the analysis in GC-MS/QQQ for the pyrethroids, we evaporated the hexane until dryness to recondition the samples in isooctane. The analytical measurement was performed by the Central Environmental Laboratory (GR-CEL) at EPFL. They have an optimized method to detect organochlorine pesticides in GC/MS and used it in several studies[1]. The target pesticides are listed in the table S1 below. We only detected tree compounds above the limit of quantification. However, since we did not add internal standard the quantification is difficult. The three compounds are: endrin, pp'DDD and pp'DDT.

Table S9 - Non-quantitative screening for OCPs

	LOQ [ng/L]	Detected in the screening (> LOQ)
Aldrin	3.9	
Chlordane alpha (cis)	3.9	
Chlordane gamma (trans)	3.9	
Dieldrin	3.9	
Endosulfan-I	3.9	
Endosulfan-II	3.9	
Endosulfan-sulfate	3.9	
Endrin	3.9	x
Endrin aldehyde	3.9	
HCB	4.5	
HCH alpha	3.9	
HCH beta	3.9	
HCH delta	3.9	
HCH gamma	3.9	
Heptachlor	3.9	
Heptachlor epoxide cis (B)	3.9	
Heptachlor epoxide trans (A)	3.9	
Methoxychlor	3.9	
Mirex	4.5	
Nonachlor trans	3.7	
Oxychlordane	3.7	
op' DDT	3.9	
pp' DDD	3.9	x
pp' DDE	3.9	
pp' DDT	3.9	x

[1] Lehmann, E., Fargues, M., Nfon Dibié, J.-J., Konaté, Y., de Alencastro, L.F., 2018. Assessment of water resource contamination by pesticides in vegetable-producing areas in Burkina Faso. Environ. Sci. Pollut. Res. 25, 3681–3694. https://doi.org/10.1007/s11356-017-0665-z
Schopfer, A., Estoppey, N., Omlin, J., Udrisard, R., Esseiva, P., De Alencastro, L.F., 2014. The Use of Passive Samplers to Reveal Industrial and Agricultural Pollution Trends in Swiss Rivers. CHIMIA 68, 778. https://doi.org/10.2533/chimia.2014.778
